# A way forward with eco evo devo: an extended theory of resource polymorphism with postglacial fishes as model systems

**DOI:** 10.1111/brv.12534

**Published:** 2019-06-19

**Authors:** Skúli Skúlason, Kevin J. Parsons, Richard Svanbäck, Katja Räsänen, Moira M. Ferguson, Colin E. Adams, Per‐Arne Amundsen, Pia Bartels, Colin W. Bean, Janette W. Boughman, Göran Englund, Jóhannes Guðbrandsson, Oliver E. Hooker, Alan G. Hudson, Kimmo K. Kahilainen, Rune Knudsen, Bjarni K. Kristjánsson, Camille A‐L. Leblanc, Zophonías Jónsson, Gunnar Öhlund, Carl Smith, Sigurður S. Snorrason

**Affiliations:** ^1^ Department of Aquaculture and Fish Biology Hólar University Sauðárkrókur, 551 Iceland; ^2^ Icelandic Museum of Natural History, Brynjólfsgata 5 Reykjavík IS‐107 Iceland; ^3^ Institute of Biodiversity, Animal Health & Comparative Medicine University of Glasgow Glasgow, G12 8QQ U.K.; ^4^ Animal Ecology, Department of Ecology and Genetics, Science for Life Laboratory Uppsala University, Norbyvägen 18D Uppsala, SE‐752 36 Sweden; ^5^ Department of Aquatic Ecology EAWAG, Swiss Federal Institute of Aquatic Science and Technology, and Institute of Integrative Biology, ETH‐Zurich, Ueberlandstrasse 133 CH‐8600 Dübendorf Switzerland; ^6^ Department of Integrative Biology University of Guelph Guelph, Ontario N1G 2W1 Canada; ^7^ Scottish Centre for Ecology and the Natural Environment, IBAHCM University of Glasgow Glasgow G12 8QQ U.K.; ^8^ Freshwater Ecology Group, Department of Arctic and Marine Biology, Faculty of Biosciences, Fisheries and Economics University of Tromsö Tromsö, N‐9037 Norway; ^9^ Department of Ecology and Environmental Science Umeå University Umeå, SE‐90187 Sweden; ^10^ Scottish Natural Heritage, Caspian House, Mariner Court, Clydebank Business Park Clydebank, G81 2NR U.K.; ^11^ Department of Integrative Biology Michigan State University East Lansing, MI 48824 U.S.A.; ^12^ Institute of Life and Environmental Sciences University of Iceland Reykjavik, 101 Iceland; ^13^ PR statistics LTD, 53 Morrison Street Glasgow, G5 8LB UK; ^14^ Inland Norway University of Applied Sciences, Department of Forestry and Wildlife Management, Campus Evenstad, Anne Evenstadvei 80 Koppang, NO‐2480 Norway; ^15^ School of Biology University of St Andrews, St. Andrews Fife, KY16 9AJ U.K.

**Keywords:** divergent evolution, epigenetics, genetics, niche construction, non‐genetic inheritance, phenotype, phenotypic plasticity, natural selection, polymorphic fishes, speciation

## Abstract

A major goal of evolutionary science is to understand how biological diversity is generated and altered. Despite considerable advances, we still have limited insight into how phenotypic variation arises and is sorted by natural selection. Here we argue that an integrated view, which merges ecology, evolution and developmental biology (eco evo devo) on an equal footing, is needed to understand the multifaceted role of the environment in simultaneously determining the development of the phenotype and the nature of the selective environment, and how organisms in turn affect the environment through eco evo and eco devo feedbacks. To illustrate the usefulness of an integrated eco evo devo perspective, we connect it with the theory of resource polymorphism (i.e. the phenotypic and genetic diversification that occurs in response to variation in available resources). In so doing, we highlight fishes from recently glaciated freshwater systems as exceptionally well‐suited model systems for testing predictions of an eco evo devo framework in studies of diversification. Studies on these fishes show that intraspecific diversity can evolve rapidly, and that this process is jointly facilitated by (*i*) the availability of diverse environments promoting divergent natural selection; (*ii*) dynamic developmental processes sensitive to environmental and genetic signals; and (*iii*) eco evo and eco devo feedbacks influencing the selective and developmental environments of the phenotype. We highlight empirical examples and present a conceptual model for the generation of resource polymorphism – emphasizing eco evo devo, and identify current gaps in knowledge.

## I. INTRODUCTION

Many key advances in evolutionary biology over the last century, such as the modern synthesis, have resulted from synergies among fields. Yet, our understanding of what drives the evolution of biological diversity is still limited – not least because we often adopt a discipline‐specific focus. For instance, the fields of evolutionary ecology and population genetics have both yielded strong empirical evidence for the role of natural selection in the evolution of biological diversity (Endler, 1986; Schluter, 2000), but they have done so from somewhat disparate perspectives. Evolutionary ecology has focused on relationships among the phenotype, environment and fitness, thus documenting selection, but rarely identifies how phenotypic variation arises (e.g. Danchin *et al*., 2011) or the agents of selection (e.g. MacColl, 2011). On the other hand, most applications of population genetics theory have focused primarily on changes in allele frequencies at loci that do not necessarily underlie the phenotypic targets of selection. Although this situation is now changing with the opportunities offered by second‐ and third‐generation sequencing (reviewed in Andrew *et al*., 2013), we still need greater insight into how phenotypic variation is generated and maintained (Hendrikse, Parsons, & Hallgrímsson, 2007; Minelli, 2015) and how it influences ecological and evolutionary processes (Pigliucci, 2008). This need is of fundamental importance because natural selection acts on the phenotype, which in turn is determined by a complex array of interacting mechanisms (Sultan, 2015).

The origins of phenotypic variation have been a focus of the field of evolutionary developmental biology (evo devo; see Table 1 for a glossary of terms used herein) (Parsons & Albertson, 2013; Moczek *et al*., 2015). Evo devo takes a ‘phenotype first’ approach in that it seeks to determine the developmental mechanisms that underlie phenotypic variation. The nature of these mechanisms is now being re‐evaluated because of the realization that nucleotide variation (genetic) is not the only source of heritable variation underlying the phenotype (reviewed in Danchin, 2013). One emerging theme is that development itself is a progenitor of phenotypic variation as it responds to environmental cues (in the present and past), thereby determining what heritable phenotypic variation is exposed to selection (West‐Eberhard, 2003; Gibson & Dworkin, 2004). The recognition that inherited variation can also arise through non‐genetic mechanisms (e.g. epigenetics) (Danchin, 2013; Bonduriansky & Day, 2018), is re‐invigorating research that seeks to understand how phenotypic variation is fuelling the capacity of populations to evolve (Kirschner & Gerhart, 1998; Hendrikse *et al*., 2007). Despite this shift in focus, the evo devo approach has only recently considered how development interacts with the environmental conditions experienced by organisms (Gilbert & Epel, 2015; Sultan, 2015).

**Table 1:** Glossary

Ecological inheritance	The legacies of change, in both biotic and abiotic environments, caused by niche‐constructing organisms to subsequent populations, which modify selection pressures on descendant organisms.
Evo devo	An integrative discipline dedicated to understanding how evolution and development reciprocally shape each other. The focus of the field is broad and encompasses various time scales. On a generation time scale, a key focus is how phenotypic variation arises from a developmental process as well as explaining its mechanistic basis.
Eco devo	The study of how ecological and developmental processes reciprocally shape each other
Eco‐evo dynamics	The study of how ecological factors interact with evolution. Research is broadly motivated but tends to focus on revealing what ecological factors determine the strength and direction of natural selection, and how evolution influences ecology.
Epigenetics	Broadly defined as the factors above the level of the genotype that contribute to developmental variation. More specifically, epigenetics focuses on the stable heritable phenotypes that result from structural changes in chromatin (e.g. DNA methylation or histone modification) without alterations in the DNA sequence itself. Such changes can be stable and cause long‐term changes in gene transcription which ultimately affect the phenotype.
Morph	A phenotypic variant within a population. Morphs can be discrete and easily identifiable, but many examples exist where phenotypic variation is subtler, and specializations are part of a continuum.
Ontogenetic plasticity	The changes phenotypes undergo during ontogeny in response to environmental cues.
Niche construction	Organism‐mediated environmental modifications that influence selection pressures on a recipient (populations of the focal species itself or other community members). A recipient can respond developmentally and evolutionarily to the environmental modification of the niche constructor. Developmental niche construction occurs when phenotypic transitions during ontogeny influence niche construction.
Phenotypic plasticity	The ability of an individual to produce different phenotypes under different environmental conditions. Often used synonymously with developmental plasticity.
Parental effects	The effect of a parent's phenotype or environment on offspring phenotype or performance. These can include paternal (e.g. *via* paternal care) and maternal effects (e.g. *via* egg size or oviposition site) and can be genetically determined and/or environmentally induced.
Resource polymorphism	The occurrence of discrete intraspecific morphs showing differential niche use, usually through discrete differences in feeding biology and habitat use.
Selection regime	The strength and type of natural selection faced by a population. Selection regimes may favour a single phenotype or divergent phenotypes and may also be considered strong while favouring different phenotypes across populations.
Transgenerational plasticity	A type of non‐genetic inheritance whereby the environment experienced by parents influences offspring reaction norms (different phenotypes expressed by the same genotype in different environments) and is manifested as a parent environment × offspring environment interaction.

Ecological conditions have traditionally been thought of as the arena within which natural selection operates. Natural selection itself does not generate heritable phenotypic variation but rather sorts it to alter phenotypic and genetic distributions across generations. Our understanding of what determines the strength and nature of selection regimes has been facilitated by the recent integration of ecological and evolutionary processes in the field of eco‐evolutionary dynamics (Hairston *et al*., 2005; Hendry, 2009, 2017). Importantly, this approach has demonstrated that ecologically driven adaptive phenotypic changes can feed back directly to ecology [e.g. population growth and ecosystem function (Thuiller *et al*., 2013; Raffard *et al*., 2019)] – illustrating the reciprocity between ecological and evolutionary processes (Metz, Nisbet, & Geritz, 1992; Post & Palkovacs, 2009; Kinnison, Hairston, & Hendry, 2015). Studies of eco‐evo dynamics recognize that substantial evolutionary changes can occur at ecological time scales (i.e. within a few generations), thereby confirming that contemporary evolutionary and ecological processes can be strongly coupled. This coupling is particularly relevant for ecosystems facing environmental change (Hendry & Kinnison, 1999; Hairston *et al*., 2005; Hendry *et al*., 2009; Matthews *et al*., 2011; Schoener, 2011).

The need for eco evo integration is further highlighted through the concept of niche construction, a process whereby an organism can influence selective environments by altering its own niche or the niches of other members of the community (Odling‐Smee *et al*., 2013; Matthews *et al*., 2014; Laland, Matthews, & Feldman, 2016). This concept is closely related to that of eco‐evolutionary feedbacks (see Post & Palkovacs, 2009; Sultan, 2015). However, a key difference between the two concepts is that research in eco‐evolutionary feedbacks to date has primarily focused on the effects of genetically inherited traits, while niche construction also includes the effects due to phenotypic plasticity (Matthews *et al*., 2014; Sultan, 2015). The niche construction concept also differs from that of the ‘extended phenotype’ because the latter is restricted to the environmental effects of genetically inherited traits (Dawkins, 1982). Niche construction theory also emphasizes (more so than eco‐evolutionary feedback) a role of inherited environments as a parallel route of inheritance (Danchin, 2013; Odling‐Smee *et al*., 2013). Niche construction can also cause plastic phenotypic responses, thereby influencing phenotypic variation available for selection and the evolution of reaction norms (Badyaev & Uller, 2009; Donohue, 2014; Moczek, 2015; Sultan, 2015; Wolinsky & Libby, 2016; Hendry, 2017). Eco evo theory has, however, largely ignored the fact that phenotypic variation is shaped by ecological conditions through development and that developmental outcomes can reciprocally influence ecological conditions (e.g. Gilbert, Bosch, & Ledon‐Rettig, 2015; Sultan, 2015). Therefore, both evo devo and eco evo require an integrated understanding of the effect of ecological variation on development (and *vice versa*) and how this affects evolution (see Lundsgaard‐Hansen *et al*., 2013; Laland *et al*., 2015).

**Figure 1:**
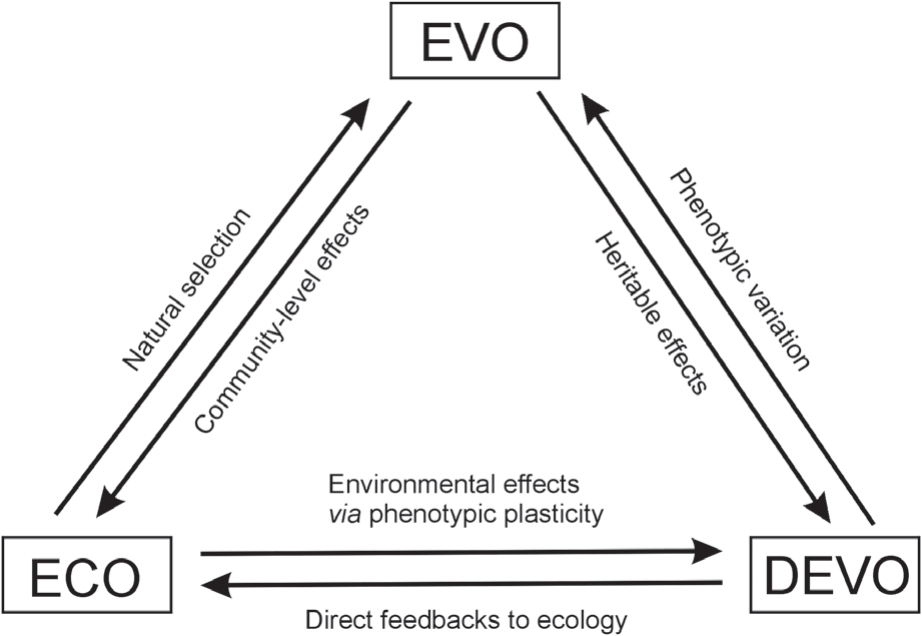
A conceptual model exploring the interactions among ecological (ECO), evolutionary (EVO) and developmental (DEVO) processes. The key interactions and pathways within this ECO EVO DEVO model can be summarized as follows. In ECO EVO, the environment influences the evolution of populations through natural selection; in EVO ECO, evolutionary responses (i.e. phenotypic changes across generations) influence ecological processes in an ecosystem (often referred to as ECO–EVO feedbacks or niche construction); in ECO DEVO, the environment affects developmental processes of individual organisms (broadly encompassing any form of individual plasticity and parental effects); in DEVO ECO, within‐generation developmental responses of individuals influence the response of populations and, subsequently, ecosystems to environmental change; in EVO DEVO, evolutionary processes across generations provide inherited signals (e.g. direct genetic and epigenetic variation) that influence phenotypic development; and in DEVO EVO, selection acts on phenotypic variation from development. In nature, ECO EVO DEVO processes interact and are likely to act dynamically, that is *via* reciprocal feedback responses.

We argue that to understand how phenotypic variation originates, evolves and feeds back on ecological processes, it is necessary to integrate the fields of evo devo and eco evo into a ‘eco evo devo’ framework where all disciplines are on an equal footing (Fig. 1). Previous discussions of the eco evo devo concept have most often considered this integration from the perspective of evo devo (see Section II). Here, we take a step forward to demonstrate how ecology (and its multitude of abiotic and biotic factors) affects organismal development, how developmental processes can in turn feed back on ecosystem‐level effects, and that these interactions can themselves evolve as well as feedback to shape the speed and direction of phenotypic evolution (Fig. 1). Given that the integration of fields often results in synergies that can lead to novel hypotheses and implementation of new methodologies, we apply the proposed eco evo devo framework to an existing theory of adaptive divergence – the theory of resource polymorphism (Skúlason & Smith, 1995).

We begin by presenting a brief historical account of the use of the term eco evo devo better to understand the development of the ideas presented in our proposed conceptual framework. We then provide an overview of resource polymorphism theory and argue that freshwater fishes inhabiting recently de‐glaciated systems (see reviews by Robinson & Wilson, 1994; Smith & Skúlason, 1996; Robinson & Schluter, 2000) are particularly well suited to the investigation of adaptive divergence using an integrated eco evo devo approach. Next, we review how studies of extrinsic factors (a focus on ecology) and intrinsic factors (a focus on development) have contributed to our understanding of the evolution of biological diversity and present specific examples from resource‐polymorphic fishes. Finally, we will come full circle and use the eco evo devo approach to update resource polymorphism theory and highlight research foci that require additional attention. We will make two key arguments: first, that ecological effects on development (as a major progenitor of phenotypic variation) and, ultimately, evolution need to become a greater focus of research on adaptive divergence (e.g. Pfennig *et al*., 2010) and, second, that adaptive divergence of populations can influence ecosystem processes not only through eco‐evo feedbacks (e.g. Post & Palkovacs, 2009; Kahilainen *et al*., 2011) but also eco‐devo feedbacks (Sultan, 2015; Matthews *et al*., 2016). Finally, we will emphasize the need to pay increased attention to how the environment can impact adaptive divergence through non‐genetic modes of inheritance (Danchin & Wagner, 2010; Danchin *et al*., 2011).

## II. THE HISTORY AND USE OF THE TERM ECO EVO DEVO

The term ‘eco evo devo’ was first put forward as a recognition that a more complete understanding of the evolution of biodiversity (in this case the morphology and phylogeny of plants) requires the integration of more than two approaches (Givnish, 2003). Subsequent use of this term comes primarily from the field of evo devo, which emphasizes that the environment (eco) plays a central role in intra‐ and intergenerational processes of phenotypic and genetic change (e.g. Moczek, 2012; Abouheif *et al*., 2014; Gilbert & Epel, 2015; Moczek, 2015). This focus on ‘eco’ has been motivated by studies of phenotypic plasticity (e.g. Gilbert *et al*., 2015; Sultan, 2015) and by the appreciation that genetic accommodation and assimilation can play roles in evolutionary change (Waddington, 1957a; West‐Eberhard, 2005). The current rise of ecological evolutionary developmental biology reflects the statement by Van Valen (1973, p. 488) that ‘*A plausible argument could be made that evolution is the control of development by ecolog*y’. The notion that the field of eco evo devo provides a framework for novel integration and organization of concepts for evolutionary theory is promoted in recent publications (Ledon‐Rettig & Pfennig, 2011; Bassaglia *et al*., 2013; Benitez, Azpeitia, & Alvarez‐Buylla, 2013; Abouheif *et al*., 2014; Gilbert *et al*., 2015; Santos *et al*., 2015; Pfennig, 2016) and many suggest that such a framework should become more widely appreciated and applied (Ghalambor, Martin, & Woods, 2015; Svensson, 2018). Our proposed eco evo devo framework is an attempt to motivate such developments.

## III. RESOURCE POLYMORPHISM IN THE CONTEXT OF ECO EVO DEVO

### (1) Resource polymorphism

Resource polymorphism is defined by the occurrence of intraspecific morphs that show differential resource use, usually through differences in feeding biology and habitat use (Skúlason & Smith, 1995; Smith & Skúlason, 1996; Pfennig & Pfennig, 2012). While ecologically mediated adaptive divergence is a central topic in evolutionary biology (Endler, 1986; Schluter, 2000; Nosil, 2012), emphasis on the role of developmental processes in generating phenotypic variation differs. For example, the predominant ‘ecological speciation’ view focuses on the evolution of reproductive isolation associated with adaptive ecological divergence (Schluter, 2000; Nosil, 2012), a narrative that often sidesteps the importance of variation generated by developmental processes. By contrast, resource polymorphism theory posits that developmentally mediated phenotypic changes can underlie adaptive change, which ultimately can facilitate reproductive isolation (Smith & Skúlason, 1996; Nonaka *et al*., 2015).

The evolution of resource polymorphism has been presented in the form of a conceptual model where divergence, and potentially speciation, can take place in the following temporal sequence (Smith & Skúlason, 1996; Skúlason, Snorrason, & Jónsson, 1999): (*i*) exploitation by a monomorphic population of a new or unexploited environment – often with high levels of intraspecific competition; (*ii*) rapid phenotypic shifts, especially in behaviour, morphology and life history, primarily mediated through phenotypic plasticity; (*iii*) divergent selection and the evolution of specialized and more distinct morphological groups, accompanied by reduced phenotypic plasticity and, finally; (*iv*) reduced gene flow and the evolution of prezygotic and potentially postzygotic reproductive isolation (Smith & Skúlason, 1996). Given that the model hypothesizes that adaptive divergence is initiated by phenotypic plasticity (a developmental phenomenon) in response to ecological variation, and that resource polymorphism has ecosystem consequences (Lundsgaard‐Hansen, Matthews, & Seehausen, 2014; Thomas *et al*., 2017), integration with the evo devo and eco evo fields, through eco evo devo is timely.

### (2) Postglacial freshwater fishes as model systems

**Figure 2:**
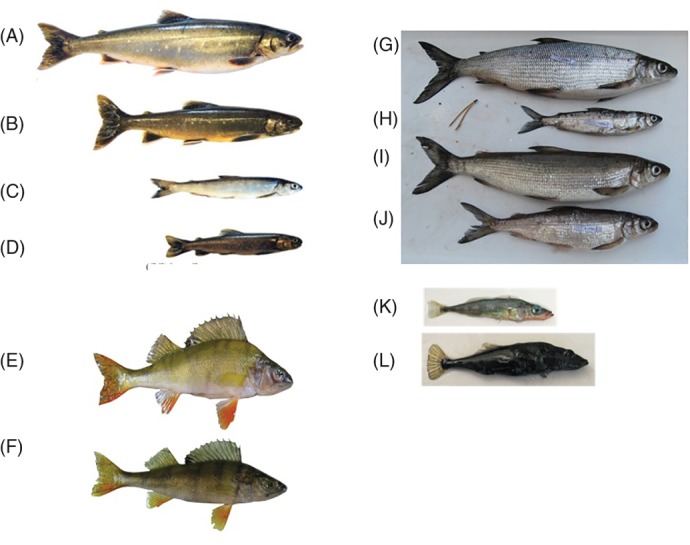
Examples of sympatric polymorphic fishes in postglacial northern lakes. (A–D) Four Arctic charr (Salmoniformes) morphs (image from Johnston *et al*., 2004), (E, F) two morphs of perch (Perciformes) (photograph: Phillip Hirsch), (G–J) four whitefish morphs (Salmoniformes) (photograph: Kimmo Kahilainen), (K, L) three‐spined stickleback (Gasterosteiformes) morph pair (photograph: Janette Boughman). See Appendix S1 and Table S1 for additional examples.

Resource polymorphism has been identified in a number of animal species (Smith & Skúlason, 1996) and has featured prominently in studies of northern freshwater fishes inhabiting recently de‐glaciated systems, including charrs (genus: *Salvelinus*), whitefish (genera: *Coregonus* and *Prosopium*), three‐spined stickleback (*Gasterosteus aculeatus*) and Eurasian perch (*Perca fluviatilis*) (Fig. 2; and see reviews in Smith & Skúlason, 1996; Skúlason *et al*., 1999; Robinson & Schluter, 2000; Snorrason & Skúlason, 2004; Hendry, 2009; Hendry *et al*., 2009). These fishes typically play a key role in their ecosystems and are particularly well‐suited model organisms for the investigation of adaptive divergence using an integrated eco evo devo approach. Their ecological, genetic and developmental tractability (*sensu* Pfennig, 2016) allows rigorous testing of key hypotheses to help establish generality in nature.

The magnitude of phenotypic divergence in postglacial freshwater fishes is associated with variation in ecological conditions (e.g. Hendry, Taylor, & McPhail, 2002; Kaeuffer *et al*., 2012), genetic connectivity (Gíslason *et al*., 1999; Lu & Bernatchez, 1999; Hendry *et al*., 2009) and phenotypic plasticity (Wimberger, 1994; Parsons *et al*., 2011). These taxa are of diverse phylogenetic origin (see Supporting information, Table S1) and have re‐colonized postglacial environments across a broad geographic area in the northern hemisphere within the last 10000–15000 years (Bernatchez & Wilson, 1998; Robinson & Schluter, 2000). Subsequently, many species have undergone rapid adaptive diversification, often within lakes where resource‐based morphs can be found at different stages of divergence and, in some cases, have evolved to form new species (Smith & Skúlason, 1996; Robinson & Schluter, 2000); Fig. 2; Appendix S1 and Table S1).

The occurrence of freshwater fishes in species‐poor systems with a well‐known history (geological, hydrological and potential anthropogenic impacts) makes them ecologically tractable. Their recent evolutionary divergence, and associated incomplete reproductive isolation (i.e. ongoing gene flow), opens a window to evolution where processes facilitating or impeding divergence can be studied along a so‐called ‘speciation continuum’ (e.g. Loh *et al*., 2008; Via, 2009). Finally, many of these species are highly amenable to both field and laboratory experimentation and now have extensive genomic resources including sequenced genomes (e.g. Christensen *et al*., 2018), thereby allowing integrated investigation of development‐ (Robinson & Parsons, 2002), evolution‐ and ecosystem‐level consequences of phenotypic variation.

The presence of substantial phenotypic differences in polymorphic fishes has often led to arguments around the genetic *versus* environmental basis of phenotypes (e.g. Nordeng, 1983). It eventually became recognized that a single species could express regional phenotypic variation, and that such variation could represent local adaptation (Schluter, 2000). Furthermore, in some populations of resource‐polymorphic fishes, plasticity‐induced phenotypic changes can parallel those typically observed in natural environments (Robinson & Parsons, 2002). For example, laboratory experiments that mimic benthic *versus* pelagic habitats in lakes commonly induce deeper *versus* shallower bodies in offspring (e.g. for Eurasian perch (Svanbäck & Eklöv, 2006). Studies have also shown how different developmental trajectories of these fishes can be shaped by interactions with the environment and internal signalling pathways (Wainwright, Osenberg, & Mittelbach, 1991; Eiríksson, Skúlason, & Snorrason, 1999; Parsons, Skúlason, & Ferguson, 2010; Leblanc *et al*., 2011; Macqueen *et al*., 2011; Parsons *et al*., 2011; Wund *et al*., 2012; Ahi *et al*., 2014; Ahi, 2016; Currey *et al*., 2017). Taken together, this combination of characteristics offers exciting opportunities to study interacting evolutionary processes in multiple highly tractable systems.

## IV. FROM THE OUTSIDE LOOKING IN: EXTRINSIC FACTORS AND THE EMERGENCE OF ECO EVO

Modern evolutionary ecology arguably arose from the need to address the problem of ‘adaptive storytelling’ (Gould & Lewontin, 1979) and, as a result, has given primacy to demonstrating natural selection as a driving force in evolution (e.g. Endler, 1986). For studies of postglacial fishes, this framework has been especially beneficial as these systems were once mostly relegated to taxonomic arguments that provided little insight into the processes that could explain patterns of inter‐ and intraspecific variation in phenotypes (Robinson & Parsons, 2002). Over the past two decades, these systems have become paradigmatic examples of adaptive phenotypic divergence and ecological speciation, largely facilitated through the evolution of resource polymorphism (Skúlason *et al*., 1999; Robinson & Schluter, 2000; Schluter, 2000; Snorrason & Skúlason, 2004) (Fig. 2; see Appendix S1 and Table S1).

Empirical evidence for the role of natural selection in diversification of polymorphic fishes has come from a combination of approaches in the field and laboratory that focus on demonstrating an association among phenotype, fitness and environment (Schluter, 2000; Kingsolver *et al*., 2001; Bolnick & Lau, 2008; Svanbäck & Persson, 2009). While these approaches do not typically identify the agents of selection (e.g. specific ecological factors) that structure local selection regimes (e.g. MacColl, 2011), they show that a range of biotic and abiotic agents of selection (and their interactions) are associated with phenotypic divergence (Robinson & Wilson, 1994; Smith & Skúlason, 1996; Robinson & Schluter, 2000; Knudsen, Amundsen, & Klemetsen, 2003; Siwertsson *et al*., 2010; Bartels *et al*., 2012; Keller & Seehausen, 2012; Woods *et al*., 2012a; Franklin *et al*., 2018). Biotic factors include low levels of interspecific but high levels of intraspecific competition, as well as interactions with prey, predators and parasites. Abiotic factors include habitat‐specific differences in water chemistry, water flow, temperature, light penetration, photoperiod, bedrock composition, and overall habitat availability through variation in lake size and lake depth.

The evolution of resource polymorphism is closely aligned with heterogeneity in the environment. As shown in three‐spined stickleback and Eurasian perch, intraspecific competition can cause polymorphism through disruptive selection (Bolnick & Lau, 2008; Svanbäck & Persson, 2009). In this scenario, disruptive selection is frequency dependent, whereby competition is more intense among ecologically similar individuals within a population, while rare types have an advantage due to reduced intraspecific competition with the most common phenotype (Bolnick, 2004; Svanbäck & Persson, 2009). Polymorphism has also arisen as the result of predation avoidance that involves trade‐offs that are habitat specific. For example, deeper bodied bluegill sunfish (*Lepomis machrochirus*) and Eurasian perch show greater survival in vegetated littoral habitats, whereas streamlined individuals survive better in open water when exposed to predation (Chipps, Dunbar, & Wahl, 2004; Svanbäck & Eklöv, 2011). Divergence of morphs is also influenced by immunological adaptations to habitat‐specific parasites (Knudsen *et al*., 2003; Eizaguirre *et al*., 2012; Karvonen *et al*., 2013) and water clarity that affects visual competence (e.g. in Eurasian perch: Bartels *et al*., 2012; Bartels *et al*., 2016). Such interactions draw attention to the dynamic relationship between eco and evo (Fig. 1).

### (1) Eco‐evo dynamics and niche construction

The development of the field of eco‐evo dynamics has been motivated by the recognition that ecological and evolutionary processes can occur at similar time scales, and by our limited understanding of what defines a selection regime (Hairston *et al*., 2005; Post & Palkovacs, 2009). Eco‐evo dynamics strive to explain both how environmental variables influence phenotypic evolution and how evolution itself acts as an agent of ecological change by creating reciprocal feedbacks between ecological and evolutionary processes (Fig. 1). These feedbacks may be strong in community‐level interactions where, for example, the evolution of predators can influence the evolution of prey (e.g. Walsh *et al*., 2012; Weis & Post, 2013). The ecological effects of intra‐ or interspecific interactions often extend beyond the focal species directly affected by those interactions, whereby evolutionary changes of a focal species may alter the environment experienced by the wider community in a given ecosystem. For example, the number of species and their traits are key predictors of many ecosystem‐level processes, such as rates of productivity, biomass sequestration and decomposition (Loreau *et al*., 2001; Schmitz, 2006). Therefore, eco‐evo feedbacks and niche construction can, in turn, influence natural selection through ‘indirect’ ecological interactions (Matthews *et al*., 2011; Schoener, 2011; Odling‐Smee *et al*., 2013; Matthews *et al*., 2014). These types of effects can be extrapolated to any number of community members and can involve dynamics that change with population demography and abiotic factors (e.g. temperature, precipitation and nutrients). The sum of these interactions can make up a selection regime, where feedbacks can change or reinforce the present conditions (e.g. *via* niche construction, see Section I).

Importantly, but frequently overlooked, the constructive contribution of species, populations or morphs to the environment can vary during ontogeny (i.e. through a devo to eco process, Fig. 1; Moczek, 2012; Donohue, 2014; Saltz & Nuzhdin, 2014; DiRienzo & Montiglio, 2016). Therefore, in addition to altering selection regimes (Best *et al*., 2017), niche construction could also change the developmental environment (Ledon‐Rettig & Pfennig, 2011; Moczek, 2015). Consequently, the environmental factors that cue development through plastic responses can also be ‘constructed’ by the ecological feedback of plastic reactions in preceding cohorts (Lundsgaard‐Hansen *et al*., 2014). Such dynamic developmental responses could alter the phenotypic variation available for natural selection and thus influence local adaptation and adaptive divergence (Pfennig *et al*., 2010; Nonaka *et al*., 2015).

In postglacial fishes, resource polymorphism can influence eco‐evo feedbacks and niche construction. For example, the presence of benthic *versus* limnetic stickleback can strongly affect prey community structure, with cascading effects on total primary production and the nature of dissolved organic matter (DOM) (Harmon *et al*., 2009). This is important because resource‐driven divergence can depend on prey community structure (Hirsch, Eklöv, & Svanbäck, 2013b), primary production (Siwertsson *et al*., 2010; Woods *et al*., 2012b) and the visual environment (Bartels *et al*., 2012; Hirsch *et al*., 2013b). Spectral properties of light transmission can further influence sexual selection and affect the extent of divergence by altering gene flow (e.g. Boughman, 2001; Candolin, Salesto, & Evers, 2007). The construction of niches by a diverging population may then eventually feed back on its selective landscape. Although understudied, such feedback loops have been observed between zooplankton and planktivorous alewife (*Alosa pseudoharengus*) and whitefish (*Coregonus lavaretus*) (Palkovacs & Post, 2009; Kahilainen *et al*., 2011). In these systems, selective feeding on different species and sizes of zooplankton induces disruptive selection on gill raker number in the fish. This in turn can affect average size and species composition of zooplankton – and thereby the selective environment for the zooplankton as well as the fish. Furthermore, phenotypic divergence of the focal fish can have a bottom‐up effect on the morphology (e.g. chain pickerel, *Esox niger*; Brodersen, Howeth, & Post, 2015) or trophic position (e.g. brown trout, *Salmo trutta*; Thomas *et al*., 2017) of their predators – which again can feed back as altered predation pressure.

Modifications of the environment by a parental generation can affect the developmental and selective environment of the offspring generation (Matthews *et al*., 2016). These ‘constructed’ conditions could prove especially relevant to evolution if they remain across generations, providing a form of ecological inheritance (Danchin, 2013; Odling‐Smee *et al*., 2013). Mesocosm experiments with polymorphic whitefish and three‐spined stickleback have shown that intra‐generation plastic phenotypic changes affect the environment (Lundsgaard‐Hansen *et al*., 2014; Matthews *et al*., 2016), influencing the selective and developmental conditions of the offspring (Sultan, 2015; see Section VI). Similarly, phenotypically plastic morphs of Eurasian perch or pumpkinseed sunfish (*Lepomis gibbosus*) (Wainwright *et al*., 1991; Parsons & Robinson, 2006; Svanbäck & Eklöv, 2006) may have environmental effects that could influence, and even reinforce, selective and developmental processes that maintain and potentially promote the evolution of further divergence (e.g. Matthews *et al*., 2016).

### (2) Resource polymorphism and ecosystem stability

Many natural populations show substantial fluctuations in density over time (Grant, 1986; Grant & Grant, 1992; Mittelbach *et al*., 1995; Smith *et al*., 1999; Klemola *et al*., 2002; Persson *et al*., 2003) that may be related to environmental factors (e.g. Grant & Grant, 1992) or consumer resource interactions (e.g. Persson *et al*., 2003). For example, summer temperature determines the growth and strength of a given year‐class in many fish species, leading to major impacts on population dynamics. Likewise, predatory or competitive interactions can drive density fluctuations over time (Townsend, Sutherland, & Perrow, 1990; Sanderson *et al*., 1999; Persson *et al*., 2003). This can lead to fluctuations in the fitness landscape (Siepielski, DiBattista, & Carlson, 2009; Svanbäck & Persson, 2009; Saether & Engen, 2015) and has the potential to play an important role in the divergence process. The effect of intraspecific heterogeneity, such as seen in polymorphic fish, on population dynamics has been little studied however (Vindenes & Langangen, 2015). In fact, population size for polymorphic fish are known from only a few lakes (Snorrason *et al*., 1992; Malinen *et al*., 2014).

Ecosystem stability or predictability will influence phenotypic trait evolution (e.g. Sultan & Spencer, 2002; Tufto, 2015) and *vice versa* (Kinnison *et al*., 2015), but also facilitates population divergence and speciation (Snorrason & Skúlason, 2004). For example, sympatric divergence of Arctic charr (*Salvelinus alpinus*) morphs in the sub‐Arctic Norwegian lake Fjellfrosvatn was characterized by temporally stable resource use, most likely reflecting predictable ecological conditions (Knudsen *et al*., 2010, 2011). Furthermore, resource‐polymorphic fish are often cannibalistic (Andersson *et al*., 2007), which may promote divergent resource specialization by stabilizing resource levels (Claessen, de Roos, & Persson, 2000). Ecosystem predictability could be enhanced during the process of diversification *via* stabilized food‐web dynamics and niche construction (Rooney & McCann, 2012; Danchin, 2013; Odling‐Smee *et al*., 2013). However, the process of divergence can itself be highly dynamic – at least until reproductive isolation between emerging species is well established. For example, introduction of zebra mussels (*Dreissena polymorpha*) to lakes has led to alterations in the visual conditions and changes in the resource base and, subsequently, to increased phenotypic divergence between littoral and pelagic Eurasian perch (Hirsch *et al*., 2013b). By contrast, eutrophication has led to breakdown of divergence in whitefish in several European lakes (Vonlanthen *et al*., 2012; Hirsch *et al*., 2013a) and in three‐spined stickleback in North America (Taylor *et al*., 2006). Once environmental conditions have returned to broadly pre‐perturbation status (e.g. before eutrophication) then divergence may re‐commence rapidly and follow similar eco‐evolutionary trajectories as before perturbation, as indicated in Lake Constance whitefish following re‐oligotrophication (Hirsch *et al*., 2013a). Such dynamism of divergence may represent what has been identified as Sysiphean evolution, where a species cycles between stages of differentiation without attaining complete reproductive isolation (McKay & Zink, 2015).

The stability or predictability of the environment is likely to interact with the underlying determinants of phenotypic variation. Theoretically, stable ecological environments should favour genetically determined, canalized phenotypes over plastic phenotypes (Hori, 1993; Scheiner, 1993; Smith, 1993), while high levels of phenotypic plasticity should be favoured in temporally unstable or spatially heterogeneous environments (Sultan & Spencer, 2002). Importantly, population density fluctuations (see above) (Svanbäck, Pineda‐Krch, & Doebeli, 2009) or other components of ecosystem stability could influence the evolution of plasticity – and plastic as well as genetically derived phenotypic change may influence population and ecosystem stability. Hence, we argue that the relationship between ecosystem stability (and instability) and the evolution of resource polymorphism needs to be examined using an eco evo devo approach, particularly in keystone species of food webs. We will return to this in Section VII when we present an extended theory of resource polymorphism.

## V. FROM THE INSIDE LOOKING OUT: INTRINSIC FACTORS AND LEVERAGING EVO DEVO FOR POSTGLACIAL FISHES

**Figure 3:**
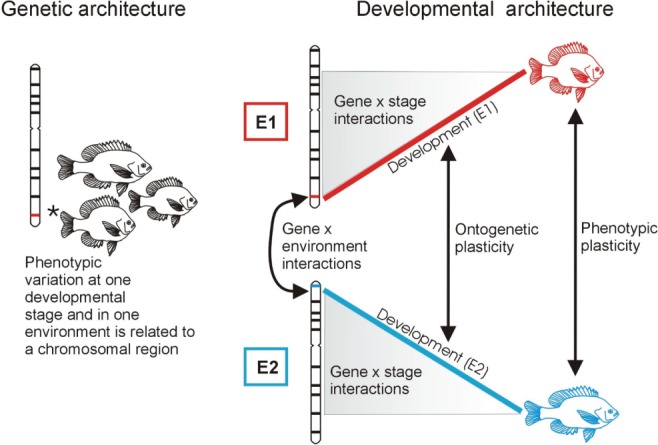
A visualization of genetic and developmental architecture. Studies on genetic architecture normally only consider relationships between genotypic variation and the phenotype at a single stage of development, and under a single set of environmental conditions. Studies on developmental architecture consider genotype/phenotype relationships that can occur across a range of environments (E1 in red and E2 in blue) and at various stages of ontogeny. Grey shaded areas represent changes in genotype/phenotype relationships that can occur over ontogenetic stages. Empirical measures of ontogenetic plasticity take into account the dynamic nature of genetic and environmental influences over developmental time (occurring from embryonic to adult stages from grey to white shaded area, respectively), which ultimately provides variation for selection at any stage. Ontogenetic plasticity accumulates to be empirically measured as phenotypic plasticity in most studies, but methodological approaches are now emerging that can account statistically for such dynamics. Environmental effects can include external ecological conditions as well as parental effects. Dynamics over ontogeny may further be influenced by epigenetic changes, which may also alter genotype/phenotype relationships and be environmentally induced.

While the ecological drivers of resource polymorphism in postglacial fishes have been extensively studied, investigations of the intrinsic factors underlying phenotypic divergence, including the genetic, developmental and physiological underpinnings, have received less attention. Yet, understanding such intrinsic factors, how they interact and are affected by the environment, is key as this will reveal if and how they are ‘seen’ by selection through their influence on phenotypic development (Houle, Govindaraju, & Omholt, 2010). This is likely to be a complex and cumulative impact stemming from both genetic variation and non‐genetic effects (e.g. epigenetic and parental effects) on development across generations (Figs 1 and 3). Apart from affecting responses to natural selection through their interaction with the environment such intrinsic factors can also influence the adaptive landscape through their feedbacks with ecological factors (Saltz & Nuzhdin, 2014). Therefore, we will next focus on research that demonstrates how developmental processes could be influenced by such dynamics and facilitate resource polymorphism in postglacial fishes**.**

### (1) Phenotypic variation results from complex genetic and environmental interactions

The developmental variation underlying phenotypes can differ markedly among populations, species and environments. For example, phenotypic plasticity has long been studied in postglacial fishes and is thought to play a significant role in their ecological divergence, especially during its early stages (e.g. Skúlason, Noakes & Snorrason, 1989; Robinson & Wilson, 1996; Svanbäck & Eklöv, 2006; Parsons *et al*., 2010, 2011; Kristjánsson *et al*., 2018). Variation in the magnitude of plasticity varies among populations (e.g. Svanbäck & Schluter, 2012; Oke *et al*., 2016) and even among morphs within a lake (Parsons *et al*., 2011). Patterns observed in morphs of Arctic charr, for instance, support the prediction of resource polymorphism theory that plasticity can be reduced in systems that are at an advanced stage of diversification (Parsons *et al*., 2011). Together, such studies of plasticity have provided some of the best evidence that phenotypic variation originates from complex interactions between the genotype and environment (Houle *et al*., 2010). Further, knowledge that plasticity is mediated through the sensitivity of developmental systems to environmental variation led to the realization that environmental sensitivity itself can evolve. This in turn has led to insights on the role of plasticity in adaptive divergence from a more mechanistic perspective (e.g. Parsons *et al*., 2016). For example, plasticity in the expression of genes implicated in the adaptive evolution of three‐spined stickleback (e.g. *PPARAa* gene involved in mitochondrial regulation) appears to facilitate colonization of freshwater environments by marine fish (Morris *et al*., 2014).

### (2) Genetic approaches towards a mechanistic understanding of adaptive phenotypes

Understanding the molecular genetic basis of phenotypic change has long been a prevalent theme in research on polymorphic postglacial fishes. Recently, molecular genetic technologies have started to provide more direct insight into the genetic mechanisms underlying resource polymorphism in postglacial fishes than was possible with quantitative genetic approaches alone. For example, quantitative trait locus (QTL) mapping continues to yield valuable information on the genomic architecture (location, number and effect size of loci) of traits involved in phenotypic divergence [e.g. Arctic charr (Küttner *et al*., 2013), three‐spined stickleback (Conte *et al*., 2015; Glazer *et al*., 2015), lake whitefish (Laporte *et al*., 2015)]. In addition, the localization of QTLs (Shapiro *et al*., 2004; Peichel, 2005) to genomic regions that have undergone divergence in natural populations has partially informed us of the genetic architecture of key traits underlying adaptive diversification (Hohenlohe *et al*., 2010; Arnegard *et al*., 2014). However, the dependency of genetic architecture on the environment and the developmental stage at which it is measured is underappreciated. This is key given that different morphs of postglacial fishes frequently develop under different environmental conditions (e.g. benthic/limnetic habitats). In Arctic charr this has resulted in substantial differences in the genetic basis of adaptive phenotypes, with the number and location of QTLs differing between fish reared in benthic or limnetic conditions (Küttner *et al*., 2014). This suggests that cryptic genetic variation is likely pervasive in postglacial fishes, with genes being ‘followers’ in evolution through their reliance on environmental conditions for their expression (Gilbert, 2001; Gilbert & Epel, 2009; Gilbert *et al*., 2015; Sultan, 2015).

While QTL studies have brought us closer to an understanding of the genetic architecture of traits, they are rarely able to pinpoint the exact nucleotide changes that take place during adaptive divergence (e.g. the predominance of gene regulatory changes in the freshwater evolution of stickleback: Jones *et al*., 2012) nor are they usually combined with functional experiments that would allow us to understand the role of specific loci in phenotypic development. Fortunately, functional genetic studies are now within reach for many postglacial fish species thanks to emerging genomic resources, including fully sequenced genomes (Berthelot *et al*., 2014; Lien *et al*., 2016; Peichel *et al*., 2017; Christensen *et al*., 2018), and the increasing ease by which genomes can be manipulated [e.g. clusters of regularly interspaced short palindromic repeats (CRISP‐R); Ran *et al*., 2013].

Transcriptomic studies have been particularly helpful in understanding processes involved in adaptive phenotypic divergence of postglacial fishes (Kitano *et al*., 2011; Hanson *et al*., 2017) where, for example, genes in bone morphogenetic protein and calcium signalling pathways are involved in the coordinated evolution of traits involved in the divergence of lake whitefish morphs (Filteau *et al*., 2013). Such functional approaches are used far less in the context of understanding developmental variation (but see Ahi *et al*., 2014, 2015) even though they could provide a direct link between environmental cues and the developmental response, as well as inform us on eco‐evolutionary feedback loops (Becks *et al*., 2012). For example, in Arctic charr differential expression of genes in the aryl hydrocarbon receptor pathway is associated with developmental variation in craniofacial traits (Ahi *et al*., 2015).

Ironically, as we learn more about the mechanistic basis of complex phenotypes and their evolution through genomic approaches, we are realizing the limitations of this strategy (Houle *et al*., 2010). The expectation that the independent evolution of similar phenotypes in similar environments corresponds to the response of similar genomic regions (Gagnaire *et al*., 2013; Perrier *et al*., 2013) and the expression of the same genes (Hanson *et al*., 2017) within them is not always upheld. For instance, in some cases the same signalling pathway, rather than the same loci, may be involved in parallel evolution as observed in the adaptation of poecilid fishes to hydrogen sulphide springs (Tobler *et al*., 2018). In addition, most genomic studies have only been able to explain a small proportion of phenotypic variance. The limitations of genomics are made even more poignant by the observation that environmental induction during development can explain similar amounts of phenotypic variation as do genomic approaches (Hu & Albertson, 2014). Quantitative genetic studies are particularly informative in estimating the relative contribution of different sources of phenotypic variation (e.g. additive genetic, maternal and environmental effects) that is available to selection in quantitative traits (Charmantier, Garant, & Kruuk, 2014). This suggests that the integration of quantitative genetic methodologies with genomic approaches can increase our ability to reveal the relative roles of developmental effects and direct genetic effects on the phenotype (Gienapp *et al*., 2017). A more systems and developmentally oriented approach that takes into account that genomic structure could impose developmental biases by limiting responses to selection (Uller *et al*., 2018) might be more fruitful for explaining the origin of diversity.

In polymorphic fishes, we are only just starting to understand the mechanistic basis of traits and how selection acts upon them within a given ecological context. Probably the best understood trait (from a mechanistic perspective) is variation in number of lateral armour plates in three‐spined stickleback. The ancestral marine type has bony armour plates along its body, while the derived freshwater types have variably reduced plate numbers (Bell & Foster, 1994; Kristjánsson, Skúlason, & Noakes, 2002b; Bell, Aguirre, & Buck, 2004). The alteration of lateral plate number by manipulating thyroid hormone levels suggests that changes in the timing of developmental events plays a key role in the origin of phenotypes associated with freshwater adaptation (Bolotovskiy *et al*., 2018). Rapid changes in the frequency and the fixation of a ‘freshwater’ allele at the ecodysplasin (EDA) locus coincide with the loss of lateral plates (Barrett, Rogers, & Schluter, 2008). The importance of this gene in freshwater adaptation is further supported by strong signatures of selection around the EDA locus in genomic comparisons of marine and freshwater fish (Roesti *et al*., 2014). The ecological significance of this trait has been demonstrated through evidence for direct selection on lateral plates independent of selection on the EDA locus (Rennison *et al*., 2015). The discrete nature of this relatively simple trait and its high tractability has enabled consummate studies on the mechanistic basis of natural phenotypic variation.

In reality, most phenotypic changes of polymorphic postglacial fishes, and adaptive radiations more generally, involve quantitative traits (e.g. body size and shape, gill raker length), which are likely to have a complex genetic basis with gene × environment interactions (Edwards, 2013; Parsons & Albertson, 2013). For example, over 130 QTLs for body shape have been detected in normal and dwarf lake whitefish (Laporte *et al*., 2015), a trait that is also influenced by environmental conditions. For most traits where we have a good understanding of why they ‘matter’ for adaptation, we usually still have little understanding of their genetic basis and developmental variation. Using complementary molecular approaches such as QTL mapping, transcriptomics and population genomics (termed a selection‐signature QTL approach, see Parsons & Albertson, 2013), combined with quantitative genetic analyses (Gienapp *et al*., 2017; Rudman *et al*., 2017), in a single study can aid in identifying genotype–phenotype–fitness relationships. However, understanding the relationship between genotype, environment and phenotype will only be achieved with better characterization of the phenotype (Houle *et al*., 2010).

### (3) Beyond genetics: phenotypic and developmental approaches to understanding adaptive variation through ‘developmental architecture’

As we understand better the genetic basis of complex phenotypic variation, we will also need to broaden our thinking to include dynamic gene–environment interactions, such as phenotypic plasticity and transgenerational (e.g. maternal and epigenetic) effects. Cues from the offspring's own environment as well as the parental phenotype (an environmental effect for the offspring; Mousseau & Fox, 1998) have the potential to alter the structure and function of the genome and influence phenotypic variation (Danchin, 2013; Smith & Ritchie, 2013; Schlichting & Wund, 2014). Phenotypic plasticity and transgenerational effects, as well as the associated non‐genetic mechanisms of inheritance (Bonduriansky & Day, 2018), are likely to be highly relevant to progressing our understanding under the eco evo devo framework (Bossdorf, Richards & Pigliucci, 2008) proposed here.

In polymorphic fish, studies on plasticity initially documented phenotypic responses to different environmental conditions (Robinson & Schluter, 2000) but became more refined by comparing the phenotypic responses of genotypes/species to environmental variation over ontogeny (e.g. Day, Pritchard, & Schluter, 1994; Parsons *et al*., 2010, 2011). Particularly relevant in the context of resource polymorphism is that diet‐induced changes in phenotype can impact foraging ability (Day *et al*., 1994; Andersson, 2003; Parsons & Robinson, 2007; Lundsgaard‐Hansen *et al*., 2013), providing a link between ecology, development, and natural selection (Fig. 1). Further, plastic phenotypic responses to diet can mirror larger patterns of trait divergence supporting the idea that plasticity provides a ‘flexible stem’ upon which further evolution can occur (Gomez‐Mestre & Buchholz, 2006; Wund *et al*., 2008, 2012; Levis, Isdaner, & Pfennig, 2018). Pre‐existing plasticity from ancestral populations can release novel phenotypic variation in response to environmental change or colonization of new habitats. According to the theory of phenotypic and genetic accommodation (West‐Eberhard, 2003, 2005), natural selection can act on this novel phenotypic variation by either refining the evolution of plastic trait responses of the emerging morphs or by promoting their developmental canalization. In its strongest form, canalization would lead to significant loss of plasticity (i.e. genetic assimilation) in relatively stable environments (West‐Eberhard, 2003, 2005; Parsons *et al*., 2011; Svanbäck & Schluter, 2012; Schlichting & Wund, 2014; Schneider & Meyer, 2017).

The genetic and developmental basis of plasticity in postglacial fishes has been addressed through the study of genes and pathways that are both evolutionarily relevant and whose expression is sensitive to environmental conditions, such as salinity and diet (e.g. McCairns & Bernatchez, 2010; Macqueen *et al*., 2011). For example, the evolution of small size of Arctic charr morphs in volcanic spring‐water systems in Iceland relative to much larger ancestors (Kapralova *et al*., 2011; Kristjánsson *et al*., 2012) is associated with the differential expression of nutritionally sensitive genes in the rapamycin (mTOR‐signalling) pathway. This altered expression leads to reduced muscle protein accretion even if the fish are reared under growth‐favouring conditions (Macqueen *et al*., 2011). Studies in other fishes have extended these ideas and provided evidence for the evolution of plasticity *via* genetic assimilation. In Malawi cichlids, the induction of benthic and limnetic (pelagic) jaw morphologies by benthic and limnetic food is associated with the effects of a regulatory locus, the patched 1 (*ptch1*) gene, which affects jaw structure through variable mediation of bone deposition around the cartilaginous precursor (Parsons *et al*., 2016). Bone deposition is associated with variable jaw movements in embryos, which influence the developmental environment leading to changes in mechanical load and ossification (Hu & Albertson, 2017). The sensitivity of the *ptch1* gene to signals from the foraging environment could then lead to selection and a decrease in environmental sensitivity through genetic assimilation (Parsons *et al*., 2016). Similarly, a study of the evolution of eye loss in the cave fish (*Astynax mexicanus*) showed that variation in eye and orbit size in surface fish was plastically increased by exposure of embryos to the low water conductivity typical of cave environments (Rohner *et al*., 2013). Increased plasticity appeared to arise from a failure of the chaperone molecule heat shock protein 90 (HSP90) to facilitate correct protein folding under stressful conditions. These findings suggest that successful colonization of caves by surface fish is facilitated by the release and subsequent selection on cryptic variation resulting in eye loss, i.e. genetic assimilation.

Transgenerational effects *via* maternal and epigenetic mechanisms can also influence diversification. Maternal effects are often prominent, and can influence the direction and speed of evolution at ecological time scales (Räsänen & Kruuk, 2007). In marine three‐spined stickleback, maternal rearing temperature affected offspring body size, and this was mediated through mitochondrial respiratory activity and the differential expression of P450 genes (Shama *et al*., 2014; Shama & Wegner, 2014). Likewise, maternal variation in egg size (correlated with yolk quantity) has large effects on progeny phenotype in salmonid fishes (e.g. Einum & Fleming, 1999; Giesing *et al*., 2011). In Arctic charr, embryos originating from small eggs tend to allocate energy towards bone development rather than body growth (Eiríksson *et al*., 1999) and are smaller and less mobile at first feeding than embryos from larger eggs (Benhaïm, Skúlason, & Hansen, 2003; Leblanc *et al*., 2011). Siblings from large eggs can also survive better than siblings from small eggs (Einum & Fleming, 1999). Such maternally driven differences in developmental trajectories can promote different trophic morphologies in offspring and contribute to the evolution of resource polymorphism. In Arctic charr, there is some evidence for maternally mediated differential expression of genes related to craniofacial development (Ahi *et al*., 2018; Beck *et al*., 2019). In brook charr (*Salvelinus fontinalis*), exposure of larger embryos to stressful environments resulted in greater plasticity and a wider developmental trajectory than in smaller embryos (Penney, Beirão, & Purchase, 2018). Although the developmental mechanisms explaining this greater plasticity are unknown in brook charr, studies of mouth‐brooding cichlids suggest that embryonic gene expression (in this case a growth hormone receptor gene *GHR*) can respond to signals effected by yolk quantity (Segers, Berishvili, & Taborsky, 2012). Such maternally driven alterations of development could lead to the persistence of maternal effects over generations if, for instance, small fish are competitively inferior and produce smaller eggs, which again leads to small size in the subsequent generation. Such egg‐size‐mediated size effects in turn should play an important role in determining what genetic variation is exposed to natural selection, and may even influence population dynamics (e.g. Beckerman *et al*., 2006; Plaistow, Lapsley, & Benton, 2006).

Epigenetics, defined as factors above the level of the genotype that contribute to developmental variation (*sensu* Waddington, 1942, 1957b), provides another potential mechanism for transgenerational plasticity. Recent epigenetics studies have focused mostly on stable heritable phenotypes that result from structural changes in chromatin (e.g. DNA methylation or histone modification) without alternations in the DNA nucleotide sequence itself (Berger *et al*., 2009; Best *et al*., 2018). Although these changes can be self‐perpetuated over generations by the phenotypic outcome of epigenetic responses (Flores, Wolschin, & Amdam, 2013), some environmentally induced epigenetic modifications are repeatable across similar environments (Le Luyer *et al*., 2017) and stably inherited across generations (Danchin, 2013). Recent studies have identified differentially methylated regions associated with adaptive phenotypic variation in postglacial fishes (Best *et al*., 2018), such as lateral plate morphs in three‐spined stickleback (Smith *et al*., 2015), migration phenotypes in rainbow trout (*Oncorhynchus mykiss*) (Baerwald *et al*., 2016) and the degree of behavioural reproductive isolation in tessellated darters (*Etheostoma olmstedi*) (Smith *et al*., 2016). However, the frequency with which environmentally induced epigenetic variation is inherited is currently unknown (Smith & Ritchie, 2013) and we need to understand how stable heritable phenotypes that result from structural changes in chromatin may feed back to, and influence, genetic variation, ecology, and evolution. As epigenetics involves an understanding of both the extrinsic and intrinsic factors that enable plastic responses, studies of polymorphic postglacial fishes under an eco evo devo framework (Fig. 1) could make a significant contribution to the field of epigenetics as a whole.

Given the above discussion, we advocate that to understand better how phenotypic variation arises and evolves it is necessary to take greater account of the diverse factors affecting development. We suggest that determining the salient ‘developmental architecture’ (rather than just the genetic architecture) under relevant ecological conditions provides an important empirical focus to help integrate eco evo devo (Fig. 3). Such studies should include the mechanistic basis of phenotypic development in nature, for instance: how molecular genetic variation interacts with internal and external environmental conditions experienced by the organism, how cells and tissues interact to achieve functional integration of the phenotype during development in a given environment (see Young & Badyaev, 2010), what the dynamics of genotype/phenotype/environment and fitness relationships are during ontogeny, and how non‐genetic inheritance mechanisms influence the evolution of the phenotype.

## IV. Empirical examples of resource polymorphism and speciation in freshwater fish from an eco evo devo perspective

In certain resource‐polymorphic fishes, aspects of the eco evo devo framework have been examined, illustrating the strength of an integrated approach.

### (1) Ecological changes facilitate phenotypic divergence: responses of perch to zebra mussel invasions

Littoral and pelagic individuals of Eurasian perch exhibit greater phenotypic divergence in lakes with invasive zebra mussels than in those without zebra mussels (Hirsch *et al*., 2013b). The presence of zebra mussels results in larger zooplankton (Idrisi *et al*., 2001; Hirsch *et al*., 2013b), a higher density of large benthic invertebrates (Ward & Ricciardi, 2007; Hirsch *et al*., 2013b) and clearer water (Higgins & Vander Zanden, 2010). Foraging on larger zooplankton leads to lower handling costs and higher energy gain for planktivorous perch (Persson, 1986) and zooplankton may also be more conspicuous to foraging fish because of increased water clarity (Ljunggren & Sandstrom, 2007). These factors favour rapid swimming in pelagic fish (Park, Lee, & Park, 2007) and, thus, a more streamlined body is advantageous (Svanbäck & Eklöv, 2003, 2004). Zebra mussels also create physical structure in the benthic habitat. This favours deep‐bodied perch which are more efficient at exploiting resources in such environments (Svanbäck & Eklöv, 2003, 2004). Furthermore, the presence of zebra mussels leads to increased densities of larger prey in benthic and pelagic habitats, causing relatively high growth rates of perch (Hirsch *et al*., 2013b). As plasticity is greater with higher growth rates, this facilitates phenotypic divergence (Olsson, Svanbäck, & Eklöv, 2006, 2007). Overall, the presence of zebra mussels changes the selective landscape through changes in resources and the visual environment (eco evo) as well as influencing the plastic response in perch through increased growth (eco devo) (Hirsch *et al*., 2013b).

### (2) Development and evolution of craniofacial diversity in charrs

Arctic charr provide a classic example of resource polymorphism with the occurrence of multiple morphs that differ in trophic morphology and size associated with resource use. Developmental studies show that differences in head and jaw shape among morphs emerge during embryonic development (Skúlason *et al*., 1989a; Ahi *et al*., 2015; Kapralova *et al*., 2015; Guðbrandsson *et al*., 2018), correlate with variable timing of bone ossification (Eiríksson *et al*., 1999) and are effected by diet (Parsons *et al*., 2011), i.e. evo devo and eco devo processes. In the closely related polymorphic Dolly Varden charr (*Salvelinus malma*), differences in adult morphology are related to variable embryonic skull ossification (Esin, Markevich, & Pichugin, 2018). In general, round head structure, blunt snout and short lower jaw – that often characterize adult benthic morphs of charr – indicate retention of embryonic morphological characters (i.e. paedomorphosis; Skúlason *et al*., 1989a). Research on craniofacial transcriptional dynamics in the progeny of benthic and pelagic Arctic charr morphs has identified a gene network, related to glucocorticoid (GC) signalling, that influences bone development and is expressed at higher levels in the benthic than the pelagic progeny (Ahi *et al*., 2014). GC signalling has been suggested to regulate Wingless/Integrated (Wnt) signalling, which is an important pathway controlling cranial bone formation (Brugmann *et al*., 2010; Ahi, 2016). Importantly, in polymorphic Malawi cichlids high levels of Wnt signalling are related to the occurrence of a short lower jaw and blunt craniofacial profile (Parsons *et al*., 2014). In cichlids, manipulation of Wnt signalling in early larvae showed that its elevation locked in the larval skull morphology resulting in an exaggerated paedomorphic benthic head shape in older progeny (Parsons *et al*., 2014). Skull development can be sensitive to environmental signals. For example, different diet‐related behaviours can mechanically influence the expression of plasticity in the relevant skeletal structures in polymorphic fish (Wainwright *et al*., 1991; Wimberger, 1994). In Arctic charr, benthic and pelagic diets promoted the development of benthic‐ and pelagic‐like head shapes in juveniles, respectively (Parsons *et al*., 2010, 2011). Furthermore, when reared on novel prey types, progeny of the pelagic morph showed greater morphological variation during ontogeny than did the progeny of the benthic morph, suggesting that the paedomorphic benthic charr had experienced stronger selection and was relatively more genetically assimilated than the pelagic morph, which was less morphologically divergent from the presumed marine ancestor (West‐Eberhard, 2003, 2005; Parsons *et al*., 2011). Elevated gene‐network activity related to skull ossification in progeny of the benthic morph could be related to such canalization (Ahi *et al*., 2014).

### (3) Evo‐eco and devo‐eco feedbacks in whitefish morphs: genetic divergence and phenotypic plasticity can affect ecosystems

Closely related but phenotypically divergent populations can have different effects on ecosystems (Harmon *et al*., 2009; Palkovacs & Post, 2009; Bassar *et al*., 2010). For example, a comparison of lakes with a single morph of European whitefish to lakes with several morphs indicates eco‐evolutionary feedbacks between whitefish and zooplankton (Kahilainen *et al*., 2011), similar to the alewife system in North America (Palkovacs & Post, 2009). Size‐selective feeding of whitefish on zooplankton in polymorphic systems in Fennoscandian postglacial lakes reduced the body size and density of zooplankton, leading to disruptive selection on gill raker number of whitefish (Kahilainen *et al*., 2011). In Lake Lucerne, Switzerland, sympatrically diverged whitefish (a benthic *Coregonus* sp. “Bodenbalchen” and a limnetic *C. zugensis*) species (Hudson, Vonlanthen, & Seehausen, 2011) are genetically differentiated in both feeding‐related morphological and behavioural traits (Vonlanthen *et al*., 2012; Lundsgaard‐Hansen *et al*., 2013) but also show notable phenotypic plasticity in foraging behaviour and efficiency (Lundsgaard‐Hansen *et al*., 2013). Direct tests of the relative contribution of genetic and plastic trait variation showed that ecosystem properties were changed through both processes (Lundsgaard‐Hansen *et al*., 2014). These studies illustrate how both evolution and ecology can affect the phenotype (evo devo and eco devo, respectively) (Vonlanthen *et al*., 2012; Lundsgaard‐Hansen *et al*., 2013) and, jointly feed back on ecosystem‐level processes (evo eco and devo eco). Thus, polymorphic postglacial fishes may simultaneously construct both their selective and developmental environments, thereby influencing their own adaptive potential both through altered selection and the expression of the phenotypic variation available for natural selection.

## VII. AN ECO EVO DEVO FRAMEWORK FOR RESOURCE POLYMORPHISM AND THE ORIGIN OF BIOLOGICAL DIVERSITY

**Figure 4:**
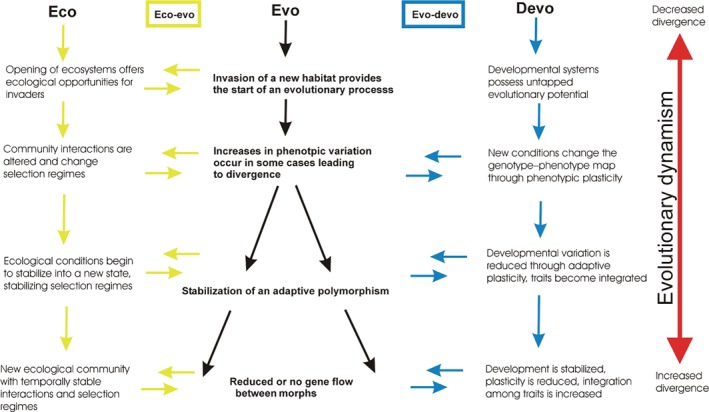
A schematic illustration of the temporal sequences of interactions that take place during the evolution of resource polymorphism within an eco evo devo framework. In this scenario, we assume colonization of a new habitat and subsequent sympatric diversification. The framework can also be applied to the more common scenario in nature where environmental change (with or without invasion of a new habitat) leads to diversification in allopatry. The series of steps under each heading (Eco, Evo, Devo) are initiated by the immigration of an organism to a new habitat (e.g. immigration of fish into myriad freshwater systems that were formed in the wake of the last glacial epoch). The Eco column shows the initial state and successive predicted ecological changes that both dictate and are influenced by evolutionary processes. This series of steps and interactions can be classified as Eco‐evo dynamics (green arrows). The Devo column shows the initial state and successive, predicted processes and changes in development that occur during adaptive divergence. These developmental processes dictate the variation that is made available for natural selection (Evo‐devo, blue arrows), but are also influenced by environmental parameters (Eco‐devo). As evolution progresses, ecological and, hence, developmental conditions are stabilized resulting in reduced phenotypic variation and more integrated phenotypes (e.g. more stable polymorphisms). Eventually ecological conditions can become stable and development canalized and traits more integrated. As a consequence of habitat matching and/or phenotype assortative mating, gene flow between morphs can be reduced or eliminated. Additionally, as indicated by the red arrow, systems are dynamic with regard to these steps. The patterns of divergence across systems can reflect any of these steps, depending on the nature of the respective processes that have shaped these patterns. Furthermore, due to environmental changes (e.g. in temperature, oxygen or light conditions, habitats, introduction of new species) plasticity can at any stage of divergence continue to provide novel phenotypic variation, such as through its diverse role over ontogeny, trait development, as well as physiological or behavioural alterations – facilitating adaptive responses of morphs. Thus, the process of divergence is dynamic and reversible, unless complete genomic isolation has evolved. The sequence of events and the processes involved with an allopatric scenario are similar to that of the sympatric scenario except that multiple morphs do not evolve as all organisms in a population are exposed to the same eco evo devo mechanisms in time and space.

We argue that using an integrative eco evo devo approach will lead to a better understanding of the emergence and role of biological diversity, and promote more focused and detailed hypotheses testing (see also Gilbert & Epel, 2015; Sultan, 2015). To illustrate the power of the eco evo devo framework, we present a conceptual model that expands the existing theory (see Section III) of resource polymorphism (Fig. 4). This model is based on a scenario where populations colonize novel and, in some cases, ecologically unstable habitats (e.g. fish invading freshwater systems emerging from the last glacial period), and undergo diversification into resource‐based morphs, which potentially results in the evolution of reproductive isolation (i.e. ecological speciation; Nosil, 2012). The model could also apply to scenarios where organisms are exposed to environments that vary temporally and spatially across their range, such that populations in different locations (i.e. allopatry) encounter different environmental conditions. This then can facilitate the evolution of geographically isolated morphs (e.g. small benthic charr in Iceland: Kapralova *et al*., 2011; Kristjánsson *et al*., 2012), whereby plasticity can provide developmental flexibility, i.e. developmental degrees of freedom (Moczek *et al*., 2011; Kristjánsson *et al*., 2018), that enables and channels divergence between populations. If such geographically separated populations come into secondary contact, the same mechanisms as in the sympatric scenario could facilitate further evolution of reproductive isolation. The novelty of our extended model of resource polymorphism rests on demonstrating the central role of the eco evo devo processes in divergence, emphasizing the dynamic relationship between ecological and developmental processes.

This extended model highlights the significance of phenotypic plasticity (at an individual and/or transgenerational level) during early divergence in novel environments (see also Wund *et al*., 2008; Schneider & Meyer, 2017; Matthews *et al*., 2018; Schwab, Casasa, & Moczek, 2019), followed by potential phenotypic canalization when environments become more structured and stable. Importantly, the model proposes that the continuous interaction of ecological and developmental features, involving eco‐evo feedbacks and niche construction, shapes the environment during the diversification process and can potentially make ecological conditions more predictable. This process is accompanied by modifications of the developmental architecture (Fig. 3) that underlies variable adaptive phenotypes and can also promote reproductive isolation through the evolution of pre‐ and post‐zygotic isolating mechanisms. Thus, the model allies with the theory of phenotypic and genetic accommodation and genetic assimilation (West‐Eberhard, 2003, 2005), where plasticity either becomes more structured or is reduced during processes of divergence, but it also emphasizes that the evolutionary process of phenotypic and genetic divergence can be highly dynamic. Thus, if environments become relatively stable over time, as would be expected for northern postglacial fishes after re‐colonization, genetic assimilation could emerge. But when ecological factors fluctuate significantly over time, typical of many environments, plastic responses may be favoured and influence performance at diverse points during ontogeny (e.g. Moczek *et al*., 2011; Parsons *et al*., 2010, Fig. 3). The responses to selection then depend on the environmental and genetic sensitivity of the underlying regulatory mechanisms (e.g. Parsons *et al*., 2016; Schneider & Meyer, 2017).

The proposed process of divergence would furthermore be affected by eco‐evo and eco‐devo feedbacks during divergence (Matthews *et al*., 2016, 2018), which could influence ecological conditions and thus selective and developmental responses (see earlier discussion and Section VI.3). The scenarios provided by this extended resource polymorphism model are supported by the observed nature and magnitude of phenotypic and genetic divergence seen in many species of postglacial fishes (see Fig. 2, Appendix S1 and Table S1), and not the least by their extensive diversity in spatial and temporal patterns of reproductive isolation, including secondary sexual contact of morphs. However, further research is needed to reveal, examine and test the diverse potential outcomes of the model.

In practical terms, rigorous tests of the different pathways of such an eco evo devo framework (Figs 1 and 4) are challenging. To facilitate such investigations, we identify some key research topics and hypotheses that are amenable to testing (Table 2). These relate to (*i*) the role of spatial and temporal variability and discreteness of resources; (*ii*) the significance of transgenerational effects; (*iii*) the role of eco‐evo feedbacks across generations and eco‐devo feedbacks during ontogeny; and (*iv*) structuring of ecosystems and developmental architecture. We strongly advocate that future research should consider explicitly the integration of these processes rather than focusing on the alternatives (such as genes *versus* environment). Thus, we need studies that explore how evolutionary and developmental processes feed back on ecosystems, both in controlled mesocosms and field experiments (e.g. Lundsgaard‐Hansen *et al*., 2014; Matthews *et al*., 2016) as well as long‐term studies in nature (Rudman *et al*., 2017), and in what way these processes affect selection regimes and developmental environments (Sultan, 2015). In line with this, studies of genotype–phenotype relationships need to be conducted more often in ecologically relevant settings, e.g. in the field or in mesocosms, rather than in standardized laboratory conditions (e.g. Küttner *et al*., 2014; Sultan, 2015). Furthermore, developmental studies should examine how environmental conditions affect gene expression, behaviour of cells during development and epigenetic patterns (including their inheritance).

**Table 2:** Key research questions on an eco evo devo framework of resource polymorphism. The following hypotheses and predictions are examples of research topics that could be examined through field and laboratory studies and experiments applying within‐ and among‐species comparisons. These research topics are naturally connected in a variety of ways differing primarily in the ‘starting point’ of the respective arguments

Research question	Background	Hypothesis	Prediction
*1. How do spatial and temporal variability and discreteness of resources interact in the generation of resource polymorphism?*	Spatial and temporal variability and discreteness of niches arise from ecological and geographical features of the environments in which resource polymorphisms occur. The environments of diverging morphs can be subtly different but also highly distinct.	The degree of spatial and temporal resource separation impacts the nature and strength of natural selection and its interplay with phenotypic plasticity and gene flow.	Temporally unstable and unpredictable (often ‘novel’) ecological conditions favour phenotypic plasticity (especially in behaviour), while more stable and predictable environments promote phenotypically segregated and less‐plastic morphs.
*2. What are the roles of epigenetic and parental effects?*	Non‐genetic inheritance mechanisms (e.g. epigenetic effects on DNA methylation) and parental effects (such as mRNA, hormones and yolk in eggs) can strongly influence phenotypic variation, and often in a highly environment‐dependent manner. Studies will need to take into account how different transgenerational effects and phenotypic plasticity, in general, operate and interact amongst themselves and with direct genetic mechanisms of inheritance.	Environmental stability influences the relative role of direct genetic and non‐genetic inheritance in determining phenotypic variation. Adaptive transgenerational plasticity will be important when environments fluctuate predictably (e.g. because of seasonality and regular population fluctuations), whereas bet‐hedging strategies will be favoured in unpredictable environments. Adaptive transgenerational plasticity allows the offspring phenotype to adaptively track favourable conditions (for example through methylation in the same genomic location), whereas bet‐hedging strategies maximize the probability of at least some offspring surviving.	Non‐genetic inheritance has significant effects during the early stages of adaptive divergence (i.e. when populations have recently invaded post‐glacial lakes and rivers) and to reinforce divergence when populations are already segregated in more distinct habitats.
*3. How do eco‐evo feedbacks and niche construction interact with eco‐devo feedbacks during ontogeny?*	Eco‐evo feedbacks and niche construction originate from phenotypic changes across generations, resulting in ecological responses that can affect both natural selection and plasticity in developmental architecture. Moreover, phenotypic changes during ontogeny can influence the within‐generation environment, leading to developmental niche construction.	(1) Niche construction through eco‐evo feedbacks of invading populations and, subsequently, diverging morphs will alter the selective environments (e.g. through predator–prey interactions and community and food‐web structuring) of the focal species or other community members (2) Niche construction through eco‐devo feedbacks, where phenotypes change due to individual or transgenerational plasticity, will influence environmental conditions.	(1) Predator–prey feedback loops, evaluated as reciprocal effects, reinforce phenotypic divergence, and community and food‐web structuring (e.g. evaluated as numbers of morphs, species and links in food webs) of diverging morphs facilitates discreteness and temporal stability of ecosystem resources, thereby strengthening divergent selection and potential reproductive isolation of morphs. (2) Eco‐devo feedbacks influence selection regimes and the nature of the environmental signalling that causes plastic responses, resulting in more structured or predictable interaction between the environment and the phenotype during ontogeny (ontogenetic niche construction)
*4. How does the structuring of ecosystems and developmental architecture interact?*	Developmental architecture (see Fig. 3) of diverging morphs and ecosystem characteristics interact at any given time. This interaction is expected to vary during divergence.	The developmental response depends on the spatial and temporal variability of ecosystems, which influences natural selection and the environmental–phenotypic interaction during development.	(1) When populations and morphs experience novel environments (e.g. following colonization), developmental systems are unstable and responsive, and (2) when environmental conditions become more predictable (e.g. when divergence of morphs is more established), developmental responses become more stable and integrated.

## VIII. CONCLUSIONS

1. We believe that the integration of multiple interacting processes into the eco evo devo framework proposed here (Fig. 1) will allow researchers to understand better how phenotypes arise and are shaped by development. Using empirical examples from polymorphic postglacial fishes we have illustrated how phenotypic change and community‐level ecological interactions can be reciprocal (Miner *et al*., 2005; Lundsgaard‐Hansen *et al.,* 2014; Matthews *et al.,* 2014). We also emphasize that phenotypic differences among individuals within and among generations are not fully dictated by differences in the DNA sequence and that a broader understanding from the perspective of developmental architecture is needed (Fig. 3) (see Danchin, 2013; Smith & Ritchie, 2013). We advocate postglacial fishes as model taxa for addressing a wide range of questions within a dynamic eco evo devo framework.
2. We emphasize that an eco evo devo approach is a powerful conceptual framework for understanding the complex integrated processes that ultimately determine the patterns of biological diversity in nature. Thus, we have discussed how natural selection is not an autonomous process that is only driven by factors external to the respective organisms, but also by intrinsic factors whereby organisms can shape their own external environment. The external factors that determine selection can often be the same as those that influence individual development through phenotypic plasticity. Thus, the snapshot of phenotype–fitness relationships we often use to measure natural selection empirically may be a subset of much broader and highly dynamic eco evo devo processes. It follows that these relationships could be viewed as evidence for coordinated variation between dynamic intrinsic developmental factors, which affect the outward phenotype, and current ecological factors. We hope that future advances in evolutionary biology will accommodate more forcefully the role of development and apply a scientific thinking that fully integrates the sub‐fields of eco, evo and devo.

## Supporting information


**Table S1.** Examples of empirical evidence for different patterns of resource polymorphism in postglacial freshwater fishes including phenotypic distribution, trait type, ecological axis of divergence, and evidence of reproductive isolation.
**Appendix S1.** Overview of resource polymorphism in fish in postglacial freshwater systems.

## References

[brv12534-bib-0001] Abouheif, E. , Fave, M. J. , Ibarraran‐Viniegra, A. S. , Lesoway, M. P. , Rafiqi, A. M. & Rajakumar, R. (2014). Eco‐evo‐devo: the time has come In Ecological Genomics: Ecology and the Evolution of Genes and Genomes *Advances in Experimental Medicine and Biology* (Volume 781, eds Landry C. R. and AubinHorth N.), pp. 107–125. Springer, New York. 10.1007/978-94-007-7347-9_6 24277297

[brv12534-bib-0002] * Adams, C. E. & Huntingford, F. A. (2002). Inherited differences in head allometry in polymorphic Arctic charr from Loch Rannoch, Scotland. Journal of Fish Biology 60, 515–520.

[brv12534-bib-0003] Ahi, E. P. (2016). Signalling pathways in trophic skeletal development and morphogenesis: insights from studies on teleost fish. Developmental Biology 420, 11–31. 27713057 10.1016/j.ydbio.2016.10.003

[brv12534-bib-0004] Ahi, E. P. , Kapralova, K. H. , Pálsson, A. , Maier, V. H. , Guðbrandsson, J. , Snorrason, S. S. , Jónsson, Z. O. & Franzdóttir, S. R. (2014). Transcriptional dynamics of a conserved gene expression network associated with craniofacial divergence in Arctic charr. EvoDevo 5, 40. 25419450 10.1186/2041-9139-5-40 PMC4240837

[brv12534-bib-0005] Ahi, E. P. , Singh, P. , Lecaudey, L. A. , Gessl, W. & Sturmbauer, C. (2018). Maternal mRNA input of growth and stress‐response‐related genes in cichlids in relation to egg size and trophic specialization. EvoDevo 9, 23. 30519389 10.1186/s13227-018-0112-3 PMC6271631

[brv12534-bib-0006] Ahi, E. P. , Steinhäuser, S. S. , Pálsson, A. , Franzdóttir, S. R. , Snorrason, S. S. , Maier, V. H. & Jónsson, Z. O. (2015). Differential expression of the aryl hydrocarbon receptor pathway associates with craniofacial polymorphism in sympatric Arctic charr. EvoDevo 6, 27. 26388986 10.1186/s13227-015-0022-6 PMC4574265

[brv12534-bib-0007] Andersson, J. (2003). Effects of diet‐induced resource polymorphism on performance in arctic charr (*Salvelinus alpinus*). Evolutionary Ecology Research 5, 213–228.

[brv12534-bib-0008] Andersson, J. , Byström, P. , Claessen, D. , Persson, L. & De Roos, A. M. (2007). Stabilization of population fluctuations due to cannibalism promotes resource polymorphism in fish. American Naturalist 169, 820–829. 10.1086/516846 17479467

[brv12534-bib-0009] Andrew, R. L. , Bernatchez, L. , Bonin, A. , Buerkle, C. A. , Carstens, B. C. , Emerson, B. C. , Garant, D. , Giraud, T. , Kane, N. C. , Rogers, S. M. , Slate, J. , Smith, H. , Sork, V. L. , Stone, G. N. , Vines, T. H. , Waits, L. , Widmer, A. & Rieseberg, L. H. (2013). A road map for molecular ecology. Molecular Ecology 22, 2605–2626. 23611646 10.1111/mec.12319

[brv12534-bib-0010] Arnegard, M. E. , McGee, M. D. , Matthews, B. , Marchinko, K. B. , Conte, G. L. , Kabir, S. , Bedford, N. , Bergek, S. , Chan, Y. F. , Jones, F. C. , Kingsley, D. M. , Peichel, C. L. & Schluter, D. (2014). Genetics of ecological divergence during speciation. Nature 511, 307–311. 24909991 10.1038/nature13301 PMC4149549

[brv12534-bib-0011] Badyaev, A. V. & Uller, T. (2009). Parental effects in ecology and evolution: mechanisms, processes and implications. Philosophical Transactions of the Royal Society B: Biological Sciences 364, 1169–1177. 10.1098/rstb.2008.0302 PMC2666689 19324619

[brv12534-bib-0012] Baerwald, M. R. , Meek, M. H. , Stephens, M. R. , Nagarajan, R. P. , Goodbla, A. M. , Tomalty, K. M. H. , Thorgaard, G. H. , May, B. & Nichols, K. M. (2016). Migration‐related phenotypic divergence is associated with epigenetic modifications in rainbow trout. Molecular Ecology 25, 1785–1800. 25958780 10.1111/mec.13231 PMC4641842

[brv12534-bib-0013] Barrett, R. D. H. , Rogers, S. M. & Schluter, D. (2008). Natural selection on a major armor gene in threespine stickleback. Science 322, 255–257. 18755942 10.1126/science.1159978

[brv12534-bib-0014] * Barrette, M. F. , Daigle, G. & Dodson, J. J. (2009). Intraspecific vicariant history and the evolution of adaptive morphological diversity in a fish species (*Osmerus mordax*). Biological Journal of the Linnean Society 97, 140–151.

[brv12534-bib-0015] Bartels, P. , Hirsch, P. , Svanbäck, R. & Eklöv, P. (2012). Water transparency drives intra‐population divergence in Eurasian perch (*Perca fluviatilis*). PLoS One 7, e43641. 10.1371/journal.pone.0043641 PMC3422328 22912895

[brv12534-bib-0016] Bartels, P. , Hirsch, P. E. , Svanbäck, R. & Eklov, P. (2016). Dissolved organic carbon reduces habitat coupling by top predators in lake ecosystems. Ecosystems 19, 955–967.

[brv12534-bib-0017] Bassaglia, Y. , Buresi, A. , Franko, D. , Andouche, A. , Baratte, S. & Bonnaud, L. (2013). *Sepia officinalis*: a new biological model for eco‐evo‐devo studies. Journal of Experimental Marine Biology and Ecology 447, 4–13.

[brv12534-bib-0018] Bassar, R. D. , Marshall, M. C. , Lopez‐Sepulcre, A. , Zandona, E. , Auer, S. K. , Travis, J. , Pringle, C. M. , Flecker, A. S. , Thomas, S. A. , Fraser, D. F. & Reznick, D. N. (2010). Local adaptation in Trinidadian guppies alters ecosystem processes. Proceedings of the National Academy of Sciences of the United States of America 107, 3616–3621. 20133670 10.1073/pnas.0908023107 PMC2840427

[brv12534-bib-0019] Beck, S. V. , Räsänen, K. , Ahi, E. P. , Kristjánsson, B. K. , Skúlason, S. , Jónsson, Z. O. & Leblanc, C. A. (2019). Gene expression in the phenotypically plastic Arctic charr (*Salvelinus alpinus*): a focus on growth and ossification at early stages of development. Evolution & Development 21, 16–30. 30474913 10.1111/ede.12275 PMC9285049

[brv12534-bib-0020] Beckerman, A. P. , Benton, T. G. , Lapsley, C. T. & Koesters, N. (2006). How effective are maternal effects at having effects? Proceedings of the Royal Society B‐Biological Sciences 273, 485–493. 10.1098/rspb.2005.3315 PMC1560202 16615217

[brv12534-bib-0021] Becks, L. , Ellner, S. P. , Jones, L. E. & Hairston, N. G. (2012). The functional genomics of an eco‐evolutionary feedback loop: linking gene expression, trait evolution, and community dynamics. Ecology Letters 15, 492–501. 22417636 10.1111/j.1461-0248.2012.01763.x

[brv12534-bib-0022] Bell, M. A. & Foster, S. A. (1994). The Evolutionary Biology of Threespine Stickleback. Oxford University Press, Oxford.

[brv12534-bib-0023] Bell, M. A. , Aguirre, W. E. & Buck, N. J. (2004). Twelve years of contemporary armor evolution in a threespine stickleback population. Evolution 58, 814–824. 15154557 10.1111/j.0014-3820.2004.tb00414.x

[brv12534-bib-0024] Benhaïm, D. , Skúlason, S. & Hansen, B. R. (2003). Behavioural variation in juvenile Arctic charr in relation to body size. Journal of Fish Biology 62, 1326–1338.

[brv12534-bib-0025] Benitez, M. , Azpeitia, E. & Alvarez‐Buylla, E. R. (2013). Dynamic models of epidermal patterning as an approach to plant eco‐evo‐devo. Current Opinion in Plant Biology 16, 11–18. 23219864 10.1016/j.pbi.2012.11.005

[brv12534-bib-0026] Berger, S. L. , Kouzarides, T. , Shiekhattar, R. & Shilatifard, A. (2009). An operational definition of epigenetics. Genes & Development 23, 781–783. 19339683 10.1101/gad.1787609 PMC3959995

[brv12534-bib-0027] * Bernatchez, L. (1997). Mitochondrial DNA analysis confirms the existence of two glacial races of rainbow smelt *Osmerus mordax* and their reproductive isolation in the St Lawrence River estuary (Quebec, Canada). Molecular Ecology 6, 73–83.

[brv12534-bib-0028] * Bernatchez, L. (2004). Ecological theory of adaptive radiation: empirical assessment from Coregonine fishes (Salmoniformes) In Evolution Illuminated: Salmon and Their Relatives (eds Hendry A. P. and Stearns S. C.), pp. 175–207. Oxford University Press, Oxford.

[brv12534-bib-0029] Bernatchez, L. & Wilson, C. C. (1998). Comparative phylogeography of Nearctic and Palearctic fishes. Molecular Ecology 7, 431–452.

[brv12534-bib-0030] * Bernatchez, L. , Chouinard, A. & Lu, G. Q. (1999). Integrating molecular genetics and ecology in studies of adaptive radiation: whitefish, *Coregonus* sp., as a case study. Biological Journal of the Linnean Society 68, 173–194.

[brv12534-bib-0031] * Bernatchez, L. , Renaut, S. , Whiteley, A. R. , Derome, N. , Jeukens, J. , Landry, L. , Lu, G. Q. , Nolte, A. W. , Østbye, K. , Rogers, S. M. & St‐Cyr, J. (2010). On the origin of species: insights from the ecological genomics of lake whitefish. Philosophical Transactions of the Royal Society B‐Biological Sciences 365, 1783–1800. 10.1098/rstb.2009.0274 PMC2871888 20439281

[brv12534-bib-0032] Berthelot, C. , Brunet, F. , Chalopin, D. , Juanchich, A. , Bernard, M. , Noël, B. , Bento, P. , Da Silva, C. , Labadie, K. , Alberti, A. , Aury, J.‐M. , Louis, A. , Dehais, P. , Bardou, P. , Montfort, J. , Klopp, C. , et al. (2014). The rainbow trout genome provides novel insights into evolution after whole‐genome duplication in vertebrates. Nature Communications 5, 3657. 10.1038/ncomms4657 PMC4071752 24755649

[brv12534-bib-0033] Best, C. , Ikert, H. , Kostyniuk, D. J. , Craig, P. M. , Navarro‐Martin, L. , Marandel, L. & Mennigen, J. A. (2018). Epigenetics in teleost fish: from molecular mechanisms to physiological phenotypes. Comparative Biochemistry and Physiology Part B: Biochemistry and Molecular Biology 224, 210–244. 10.1016/j.cbpb.2018.01.006 29369794

[brv12534-bib-0034] Best, R. J. , Anaya‐Rojas, J. M. , Leal, M. C. , Schmid, D. W. , Seehausen, O. & Matthews, B. (2017). Transgenerational selection driven by divergent ecological impacts of hybridizing lineages. Nature Ecology & Evolution 1, 1757–1765. 28963514 10.1038/s41559-017-0308-2

[brv12534-bib-0035] Bolnick, D. I. (2004). Can intraspecific competition drive disruptive selection? An experimental test in natural populations of sticklebacks. Evolution 58, 608–618. 15119444

[brv12534-bib-0036] Bolnick, D. I. & Lau, O. L. (2008). Predictable patterns of disruptive selection in stickleback in postglacial lakes. American Naturalist 172, 1–11. 10.1086/587805 18452402

[brv12534-bib-0037] Bolotovskiy, A. A. , Levina, M. A. , DeFaveri, J. , Merilä, J. & Levin, B. A. (2018). Heterochronic development of lateral plates in the three‐spined stickleback induced by thyroid hormone level alterations. PLoS One 13, e0194040. 10.1371/journal.pone.0194040 PMC5844557 29522555

[brv12534-bib-0038] Bonduriansky, R. & Day, T. (2018). Extended Heredity. A New Understanding of Inheritance and Evolution. Princeton University Press, Princeton.

[brv12534-bib-0039] * Bossdorf, O. , Richards, C. L. & Pigliucci, M. (2008). Epigenetics for ecologists. Ecology Letters 11, 106–115. 18021243 10.1111/j.1461-0248.2007.01130.x

[brv12534-bib-0040] Boughman, J. W. (2001). Divergent sexual selection enhances reproductive isolation in sticklebacks. Nature 411, 944–948. 11418857 10.1038/35082064

[brv12534-bib-0041] * Bourke, P. , Magnan, P. & Rodriguez, M. A. (1997). Individual variations in habitat use and morphology in brook charr. Journal of Fish Biology 51, 783–794.

[brv12534-bib-0042] * Bradbury, I. R. , Coulson, M. W. , Cook, A. M. & Bentzen, P. (2010). Evidence for divergence and adaptive isolation in post‐glacially derived bimodal allopatric and sympatric rainbow smelt populations. Biological Journal of the Linnean Society 101, 583–594.

[brv12534-bib-0043] Brodersen, J. , Howeth, J. G. & Post, D. M. (2015). Emergence of a novel prey life history promotes contemporary sympatric diversification in a top predator. Nature Communications 6, 8115. 10.1038/ncomms9115 26365323

[brv12534-bib-0044] Brugmann, S. A. , Powder, K. E. , Young, N. M. , Goodnough, L. H. , Hahn, S. M. , James, A. W. , Helms, J. A. & Lovett, M. (2010). Comparative gene expression analysis of avian embryonic facial structures reveals new candidates for human craniofacial disorders. Human Molecular Genetics 19, 920–930. 20015954 10.1093/hmg/ddp559 PMC2816616

[brv12534-bib-0045] * Byström, P. & Andersson, J. (2005). Size‐dependent foraging capacities and intercohort competition in an ontogenetic omnivore (Arctic char). Oikos 110, 523–536.

[brv12534-bib-0046] Candolin, U. , Salesto, T. & Evers, M. (2007). Changed environmental conditions weaken sexual selection in sticklebacks. Journal of Evolutionary Biology 20, 233–239. 17210016 10.1111/j.1420-9101.2006.01207.x

[brv12534-bib-0047] Charmantier, A. , Garant, D. & Kruuk, L. E. B. (2014). Quantitative Genetics in the Wild. Oxford University Press, Oxford.

[brv12534-bib-0048] * Chavarie, L. , Howland, K. L. & Tonn, W. M. (2013). Sympatric polymorphism in lake Trout: the coexistence of multiple shallow‐water morphotypes in Great Bear Lake. Transactions of the American Fisheries Society 142, 814–823.

[brv12534-bib-0049] Chipps, S. R. , Dunbar, J. A. & Wahl, D. H. (2004). Phenotypic variation and vulnerability to predation in juvenile bluegill sunfish (*Lepomis macrochirus*). Oecologia 138, 32–38. 14517677 10.1007/s00442-003-1396-z

[brv12534-bib-0050] Christensen, K. A. , Rondeau, E. B. , Minkley, D. R. , Leong, J. S. , Nugent, C. M. , Danzmann, R. G. , Ferguson, M. M. , Stadnik, A. , Devlin, R. H. , Muzzerall, R. , Edwards, M. , Davidson, W. S. & Koop, B. F. (2018). The Arctic charr (*Salvelinus alpinus*) genome and transcriptome assembly. PLoS One 13, e0204076. 10.1371/journal.pone.0204076 PMC6136826 30212580

[brv12534-bib-0051] Claessen, D. , de Roos, A. M. & Persson, L. (2000). Dwarfs and giants: cannibalism and competition in size‐structured populations. American Naturalist 155, 219–237. 10.1086/303315 10686162

[brv12534-bib-0052] * Colborne, S. F. , Garner, S. R. , Longstaffe, F. J. & Neff, B. D. (2016). Assortative mating but no evidence of genetic divergence in a species characterized by a trophic polymorphism. Journal of Evolutionary Biology 29, 633–644. 26688005 10.1111/jeb.12812

[brv12534-bib-0053] Conte, G. L. , Arnegard, M. E. , Best, J. , Chan, Y. G. F. , Jones, F. C. , Kingsley, D. M. , Schluter, D. & Peichel, C. L. (2015). Extent of QTL reuse during repeated phenotypic divergence of sympatric threespine stickleback. Genetics 201, 1189–U730. 26384359 10.1534/genetics.115.182550 PMC4649644

[brv12534-bib-0054] * Crespel, A. , Dupont‐Prinet, A. , Bernatchez, L. , Claireaux, G. , Tremblay, R. & Audet, C. (2017). Divergence in physiological factors affecting swimming performance between anadromous and resident populations of brook charr *Salvelinus fontinalis* . Journal of Fish Biology 90, 2170–2193. 28317121 10.1111/jfb.13300

[brv12534-bib-0055] Currey, M. C. , Bassham, S. , Perry, S. & Cresko, W. A. (2017). Developmental timing differences underlie armor loss across threespine stickleback populations. Evolution & Development 19, 231–243. 29115024 10.1111/ede.12242

[brv12534-bib-0056] * Curry, R. A. , Currie, S. L. , Bernatchez, L. & Saint‐Laurent, R. (2004). The rainbow smelt, *Osmerus mordax*, complex of Lake Utopia: threatened or misunderstood? Environmental Biology of Fishes 69, 153–166.

[brv12534-bib-0057] Danchin, E. (2013). Avatars of information: towards an inclusive evolutionary synthesis. Trends in Ecology & Evolution 28, 351–358. 23540765 10.1016/j.tree.2013.02.010

[brv12534-bib-0058] Danchin, E. & Wagner, R. H. (2010). Inclusive heritability: combining genetic and non‐genetic information to study animal behavior and culture. Oikos 119, 210–218.

[brv12534-bib-0059] Danchin, E. , Charmantier, A. , Champagne, F. A. , Mesoudi, A. , Pujol, B. & Blanchet, S. (2011). Beyond DNA: integrating inclusive inheritance into an extended theory of evolution. Nature Reviews Genetics 12, 475–486. 10.1038/nrg3028 21681209

[brv12534-bib-0060] Dawkins, R. (1982). The Extended Phenotype. Oxford University Press, Oxford.

[brv12534-bib-0061] Day, T. , Pritchard, J. & Schluter, D. (1994). A comparison of 2 sticklebacks. Evolution 48, 1723–1734. 28568405 10.1111/j.1558-5646.1994.tb02208.x

[brv12534-bib-0062] * Delling, B. , Palm, S. , Palkopoulou, E. & Prestegaard, T. (2014). Genetic signs of multiple colonization events in Baltic ciscoes with radiation into sympatric spring‐ and autumn‐spawners confined to early postglacial arrival. Ecology and Evolution 4, 4346–4360. 25540695 10.1002/ece3.1299 PMC4267872

[brv12534-bib-0063] DiRienzo, N. & Montiglio, P. O. (2016). The contribution of developmental experience vs. condition to life history, trait variation and individual differences. Journal of Animal Ecology 85, 915–926. 26937627 10.1111/1365-2656.12512 PMC5129655

[brv12534-bib-0064] Donohue, K. (2014). Why ontogeny matters during adaptation: developmental niche construction and pleiotrophy across the life cycle in *Arabidopsis thaliana* . Evolution 68, 32–47. 24117399 10.1111/evo.12284

[brv12534-bib-0065] Edwards, S. V. (2013). Next‐generation QTL mapping: crowdsourcing SNPs, without pedigrees. Molecular Ecology 22, 3885–3887. 24058928 10.1111/mec.12401

[brv12534-bib-0066] * Ehlinger, T. J. (1990). Habitat choice and phenotype‐limited feeding efficiency in Bluegill ‐ individual‐differences and trophic polymorphism. Ecology 71, 886–896.

[brv12534-bib-0067] * Ehlinger, T. J. & Wilson, D. S. (1988). Complex foraging polymorphism in Bluegill sunfish. Proceedings of the National Academy of Sciences of the United States of America 85, 1878–1882. 16578831 10.1073/pnas.85.6.1878 PMC279884

[brv12534-bib-0068] Einum, S. & Fleming, I. A. (1999). Maternal effects of egg size in brown trout (*Salmo trutta*): norms of reaction to environmental quality. Proceedings of the Royal Society B‐Biological Sciences 266, 2095–2100.

[brv12534-bib-0069] Eiríksson, G. M. , Skúlason, S. & Snorrason, S. S. (1999). Heterochrony in skeletal development and body size in progeny of two morphs of Arctic charr from Thingvallavatn, Iceland. Journal of Fish Biology 55, 175–185.

[brv12534-bib-0070] Eizaguirre, C. , Lenz, T. L. , Kalbe, M. & Milinski, M. (2012). Divergent selection on locally adapted major histocompatibility complex immune genes experimentally proven in the field. Ecology Letters 15, 723–731. 22583762 10.1111/j.1461-0248.2012.01791.x PMC3440595

[brv12534-bib-0071] * Eklöv, P. & Svanbäck, R. (2006). Predation risk influences adaptive morphological variation in fish populations. American Naturalist 167, 440–452. 10.1086/499544 16673351

[brv12534-bib-0072] Endler, J. A. (1986). Natural Selection in the Wild. Princeton University Press, Princeton, NJ.

[brv12534-bib-0073] * Eshenroder, R. L. , Vecsei, P. , Gorman, O. T. , Yule, D. L. , Pratt, T. C. , Mandrak, N. E. , Bunnell, D. B. & Muir, A. M. (2016). Ciscoes (*Coregonus*, subgenus *Leucichthys*) of the Laurentian Great Lakes and Lake Nipigon Great Lakes Fishery Commission, pp. 16‐01. Miscellaneous Publication, Ann Arbor, Michigan.

[brv12534-bib-0074] Esin, E. V. , Markevich, G. N. & Pichugin, M. Y. (2018). Juvenile divergence in adaptive traits among seven sympatric fish ecomorphs arises before moving to different lacustrine habitats. Journal of Evolutionary Biology 31, 1018–1034. 29672982 10.1111/jeb.13283

[brv12534-bib-0075] * Faulks, L. , Svanbäck, R. , Eklöv, P. & Östman, Ö. (2015). Genetic and morphological divergence along the littoral‐pelagic axis in two common and sympatric fishes: perch, *Perca fluviatilis* (Percidae) and roach, *Rutilus rutilus* (Cyprinidae). Biological Journal of the Linnean Society 114, 929–940.

[brv12534-bib-0076] Filteau, M. , Pavey, S. A. , St‐Cyr, J. & Bernatchez, L. (2013). Gene coexpression networks reveal key drivers of phenotypic divergence in lake whitefish. Molecular Biology and Evolution 30, 1384–1396. 23519315 10.1093/molbev/mst053

[brv12534-bib-0077] Flores, K. B. , Wolschin, F. & Amdam, G. V. (2013). The Role of Methylation of DNA in Environmental Adaptation. Integrative and Comparative Biology 53, 359–372. 23620251 10.1093/icb/ict019 PMC3710460

[brv12534-bib-0078] Franklin, O. D. , Skúlason, S. , Morrissey, M. B. & Ferguson, M. M. (2018). Natural selection for body shape in resource polymorphic Icelandic Arctic charr. Journal of Evolutionary Biology 31, 1498–1512. 29961959 10.1111/jeb.13346

[brv12534-bib-0079] Gagnaire, P. A. , Pavey, S. A. , Normandeau, E. & Bernatchez, L. (2013). The genetic architecture of reproductive isolation during speciation‐with‐gene‐flow in lake whitefish species pairs assessed by RAD sequencing. Evolution 67, 2483–2497. 24033162 10.1111/evo.12075

[brv12534-bib-0080] Ghalambor, C. K. , Martin, L. B. & Woods, H. A. (2015). Plasticity, complexity and the individual In Integrative Organismal Biology (eds Martin L. B., Ghalambor C. K. and Woods H. A.). John Whiley & Sons, Hoboken.

[brv12534-bib-0081] Gibson, G. & Dworkin, I. (2004). Uncovering cryptic genetic variation. Nature Reviews Genetics 5, 681–U11. 10.1038/nrg1426 15372091

[brv12534-bib-0082] Gienapp, P. , Fior, S. , Guillaume, F. , Lasky, J. R. , Sork, V. L. & Csilléry, K. (2017). Genomic quantitative genetics to study evolution in the wild. Trends in Ecology & Evolution 32, 897–908. 29050794 10.1016/j.tree.2017.09.004

[brv12534-bib-0083] Giesing, E. R. , Suski, C. D. , Warner, R. E. & Bell, A. M. (2011). Female sticklebacks transfer information via eggs: effects of maternal experience with predators on offspring. Proceedings of the Royal Society B‐Biological Sciences 278, 1753–1759. 10.1098/rspb.2010.1819 PMC3081764 21068041

[brv12534-bib-0084] Gilbert, S. F. (2001). Ecological developmental biology: developmental biology meets the real world. Developmental Biology 233, 1–12. 11319853 10.1006/dbio.2001.0210

[brv12534-bib-0085] Gilbert, S. F. & Epel, D. (2009). Ecological Developmental Biology: Integrating Epigenetics, Medicine, and Evolution. Sinauer Associates, Sunderland.

[brv12534-bib-0086] Gilbert, S. F. & Epel, D. (2015). Ecological Developmental Biology: The Environmental Regulation of Development, Health, and Evolution. Sinauer Associates, Sunderland.

[brv12534-bib-0087] Gilbert, S. F. , Bosch, T. C. G. & Ledon‐Rettig, C. (2015). Eco‐Evo‐Devo: developmental symbiosis and developmental plasticity as evolutionary agents. Nature Reviews Genetics 16, 611–622. 10.1038/nrg3982 26370902

[brv12534-bib-0088] Gíslason, D. , Ferguson, M. , Skúlason, S. & Snorrason, S. S. (1999). Rapid and coupled phenotypic and genetic divergence in Icelandic Arctic char (*Salvelinus alpinus*). Canadian Journal of Fisheries and Aquatic Sciences 56, 2229–2234.

[brv12534-bib-0089] Givnish, T. J. (2003). How a better understanding of adaptations can yield better use of morphology in plant systematics: toward Eco‐Evo‐Devo In Deep Morphology: Toward a Renaissance of Morphology in Plant Systematics *Regnum Vegetabile* (Volume 141, eds Stuessy T. F., Mayer V. and Horandl E.), pp. 273–295. A R G Gantner Verlag K G, Koenigstein.

[brv12534-bib-0090] Glazer, A. M. , Killingbeck, E. E. , Mitros, T. , Rokhsar, D. S. & Miller, C. T. (2015). Genome assembly improvement and mapping convergently evolved skeletal traits in sticklebacks with genotyping‐by‐sequencing. G3‐Genes Genomes Genetics 5, 1463–1472. 10.1534/g3.115.017905 PMC4502380 26044731

[brv12534-bib-0091] Gomez‐Mestre, I. & Buchholz, D. R. (2006). Developmental plasticity mirrors differences among taxa in spadefoot toads linking plasticity and diversity. Proceedings of the National Academy of Sciences of the United States of America 103, 19021–19026. 17135355 10.1073/pnas.0603562103 PMC1748170

[brv12534-bib-0092] Gould, S. J. & Lewontin, R. C. (1979). The spandrels of San‐Marco and the Panglossian paradigm ‐ a critique of the adaptationist program. Proceedings of the Royal Society Series B‐Biological Sciences 205, 581–598. 10.1098/rspb.1979.0086 42062

[brv12534-bib-0093] * Gowell, C. P. , Quinn, T. P. & Taylor, E. B. (2012). Coexistence and origin of trophic ecotypes of pygmy whitefish, *Prosopium coulterii*, in a south‐western Alaskan lake. Journal of Evolutionary Biology 25, 2432–2448. 23110688 10.1111/jeb.12011

[brv12534-bib-0094] Grant, P. R. (1986). Ecology and Evolution of Darwin's Finches. Princeton University Press, Princeton.

[brv12534-bib-0095] Grant, P. R. & Grant, B. R. (1992). Demography and the genetically effective sizes of 2 populations of Darwin's finches. Ecology 73, 766–784.

[brv12534-bib-0096] Guðbrandsson, J. , Franzdóttir, S. R. , Kristjánsson, B. K. , Ahi, E. P. , Maier, V. H. , Kapralova, K. H. , Snorrason, S. S. , Jónsson, Z. O. & Pálsson, A. (2018). Differential gene expression during early development in recently evolved and sympatric Arctic charr morphs. PeerJ 6, e4345. 29441236 10.7717/peerj.4345 PMC5807978

[brv12534-bib-0097] Hairston, N. G. , Ellner, S. P. , Geber, M. A. , Yoshida, T. & Fox, J. A. (2005). Rapid evolution and the convergence of ecological and evolutionary time. Ecology Letters 8, 1114–1127.

[brv12534-bib-0098] * Hansen, M. J. , Nate, N. A. , Krueger, C. C. , Zimmerman, M. S. , Kruckman, H. G. & Taylor, W. W. (2012). Age, growth, survival, and maturity of Lake trout morphotypes in Lake Mistassini, Quebec. Transactions of the American Fisheries Society 141, 1492–1503.

[brv12534-bib-0099] Hanson, D. , Hu, J. , Hendry, A. P. & Barrett, R. D. H. (2017). Heritable gene expression differences between lake and stream stickleback include both parallel and antiparallel components. Heredity 119, 339–348. 28832577 10.1038/hdy.2017.50 PMC5637370

[brv12534-bib-0100] Harmon, L. J. , Matthews, B. , Des Roches, S. , Chase, J. M. , Shurin, J. B. & Schluter, D. (2009). Evolutionary diversification in stickleback affects ecosystem functioning. Nature 458, 1167–1170. 19339968 10.1038/nature07974

[brv12534-bib-0101] * Harrod, C. , Mallela, J. & Kahilainen, K. K. (2010). Phenotype‐environment correlations in a putative whitefish adaptive radiation. Journal of Animal Ecology 79, 1057–1068. 20487087 10.1111/j.1365-2656.2010.01702.x

[brv12534-bib-0102] * Helland, I. P. , Harrod, C. , Freyhof, J. & Mehner, T. (2008). Co‐existence of a pair of pelagic planktivorous coregonid fishes. Evolutionary Ecology Research 10, 373–390.

[brv12534-bib-0103] * Helland, I. P. , Vøllestad, L. A. , Freyhof, J. & Mehner, T. (2009). Morphological differences between two ecologically similar sympatric fishes. Journal of Fish Biology 75, 2756–2767. 20738521 10.1111/j.1095-8649.2009.02476.x

[brv12534-bib-0104] Hendrikse, J. L. , Parsons, T. E. & Hallgrímsson, B. (2007). Evolvability as the proper focus of evolutionary developmental biology. Evolution & Development 9, 393–401. 17651363 10.1111/j.1525-142X.2007.00176.x

[brv12534-bib-0105] Hendry, A. P. (2009). Ecological speciation! Or the lack thereof? Canadian Journal of Fisheries and Aquatic Sciences 66, 1383–1398.

[brv12534-bib-0106] Hendry, A. P. (2017). Eco‐Evolutionary Dynamics. Princeton University Press, Princeton and Oxford.

[brv12534-bib-0107] Hendry, A. P. & Kinnison, M. T. (1999). Perspective: the pace of modern life: measuring rates of contemporary microevolution. Evolution 53, 1637–1653. 28565449 10.1111/j.1558-5646.1999.tb04550.x

[brv12534-bib-0108] Hendry, A. P. , Bolnick, D. I. , Berner, D. & Peichel, C. L. (2009). Along the speciation continuum in sticklebacks. Journal of Fish Biology 75, 2000–2036. 20738669 10.1111/j.1095-8649.2009.02419.x

[brv12534-bib-0109] Hendry, A. P. , Taylor, E. B. & McPhail, J. D. (2002). Adaptive divergence and the balance between selection and gene flow: lake and stream stickleback in the Misty system. Evolution 56, 1199–1216. 12144020 10.1111/j.0014-3820.2002.tb01432.x

[brv12534-bib-0110] Higgins, S. N. & Vander Zanden, M. J. (2010). What a difference a species makes: a meta‐analysis of dreissenid mussel impacts on freshwater ecosystems. Ecological Monographs 80, 179–196.

[brv12534-bib-0111] Hirsch, P. E. , Eckmann, R. , Oppelt, C. & Behrmann‐Godel, J. (2013a). Phenotypic and genetic divergence within a single whitefish form ‐ detecting the potential for future divergence. Evolutionary Applications 6, 1119–1132. 24478795 10.1111/eva.12087 PMC3901543

[brv12534-bib-0112] Hirsch, P. E. , Eklöv, P. & Svanbäck, R. (2013b). Indirect trophic interactions with an invasive species affect phenotypic divergence in a top consumer. Oecologia 172, 245–256. 23463242 10.1007/s00442-013-2611-1

[brv12534-bib-0113] Hohenlohe, P. A. , Bassham, S. , Etter, P. D. , Stiffler, N. , Johnson, E. A. & Cresko, W. A. (2010). Population genomics of parallel adaptation in threespine stickleback using sequenced RAD tags. PLOS Genetics 6, 23. 10.1371/journal.pgen.1000862 PMC2829049 20195501

[brv12534-bib-0114] Hori, M. (1993). Frequency‐dependent natural‐selection in the handedness of scale‐eating cichlid fish. Science 260, 216–219. 17807183 10.1126/science.260.5105.216

[brv12534-bib-0115] Houle, D. , Govindaraju, D. R. & Omholt, S. (2010). Phenomics: the next challenge. Nature Reviews Genetics 11, 855–866. 10.1038/nrg2897 21085204

[brv12534-bib-0116] Hu, Y. & Albertson, R. C. (2014). Hedgehog signaling mediates adaptive variation in a dynamic functional system in the cichlid feeding apparatus. Proceedings of the National Academy of Sciences 111, 8530–8534. 10.1073/pnas.1323154111 PMC4060705 24912175

[brv12534-bib-0117] Hu, Y. N. & Albertson, R. C. (2017). Baby fish working out: an epigenetic source of adaptive variation in the cichlid jaw. Proceedings of the Royal Society B‐Biological Sciences 284, 8. 10.1098/rspb.2017.1018 PMC5563811 28768892

[brv12534-bib-0118] Hudson, A. G. , Vonlanthen, P. & Seehausen, O. (2011). Rapid parallel adaptive radiations from a single hybridogenic ancestral population. Proceedings of the Royal Society B‐Biological Sciences 278, 58–66. 10.1098/rspb.2010.0925 PMC2992718 20685705

[brv12534-bib-0119] Idrisi, N. , Mills, E. L. , Rudstam, L. G. & Stewart, D. J. (2001). Impact of zebra mussels (*Dreissena polymorpha*) on the pelagic lower trophic levels of Oneida Lake, New York. Canadian Journal of Fisheries and Aquatic Sciences 58, 1430–1441.

[brv12534-bib-0120] * Ingram, T. , Svanbäck, R. , Kraft, N. J. B. , Kratina, P. , Southcott, L. & Schluter, D. (2012). Intraguild predation drives evolutionary niche shift in threespine stickleback. Evolution 66, 1819–1832. 22671549 10.1111/j.1558-5646.2011.01545.x

[brv12534-bib-0121] * Jastrebski, C. J. & Robinson, B. W. (2004). Natural selection and the evolution of replicated trophic polymorphisms in pumpkinseed sunfish (*Lepomis gibbosus*). Evolutionary Ecology Research 6, 285–305.

[brv12534-bib-0122] Johnston, I. A. , Abercromby, M. , Vieira, V. L. A. , Sigursteindóttir, R. J. , Kristjánsson, B. K. , Sibthorpe, D. & Skúlason, S. (2004). Rapid evolution of muscle fibre number in post‐glacial populations of Arctic charr *Salvelinus alpinus* . Journal of Experimental Biology 207, 4343–4360. 15557021 10.1242/jeb.01292

[brv12534-bib-0123] Jones, F. C. , Grabherr, M. G. , Chan, Y. F. , Russell, P. , Mauceli, E. , Johnson, J. , Swofford, R. , Pirun, M. , Zody, M. C. , White, S. , Birney, E. , Searle, S. , Schmutz, J. , Grimwood, J. , Dickson, M. C. , et al. (2012). The genomic basis of adaptive evolution in threespine sticklebacks. Nature 484, 55–61. 22481358 10.1038/nature10944 PMC3322419

[brv12534-bib-0124] Kaeuffer, R. , Peichel, C. L. , Bolnick, D. I. & Hendry, A. P. (2012). Parallel and nonparallel aspects of ecological, phenotypic, and genetic divergence across replicate population pairs of lake and stream stickleback. Evolution 66, 402–418. 22276537 10.1111/j.1558-5646.2011.01440.x PMC4499166

[brv12534-bib-0125] * Kahilainen, K. & Lehtonen, H. (2002). Brown trout (*Salmo trutta* L.) and Arctic charr (*Salvelinus alpinus* L.) as predators on three sympatric whitefish (*Coregonus lavaretus* L.) forms in the subarctic Lake Muddusjarvi. Ecology of Freshwater Fish 11, 158–167.

[brv12534-bib-0126] * Kahilainen, K. & Østbye, K. (2006). Morphological differentiation and resource polymorphism in three sympatric whitefish *Coregonus lavaretus* (L.) forms in a subarctic lake. Journal of Fish Biology 68, 63–79.

[brv12534-bib-0127] Kahilainen, K. K. , Siwertsson, A. , Gjelland, K. Ø. , Knudsen, R. , Bøhn, T. & Amundsen, P. A. (2011). The role of gill raker number variability in adaptive radiation of coregonid fish. Evolutionary Ecology 25, 573–588.

[brv12534-bib-0128] Kapralova, K. H. , Jónsson, Z. O. , Pálsson, A. , Franzdóttir, S. R. , Le Deuff, S. , Kristjánsson, B. K. & Snorrason, S. S. (2015). Bones in motion: ontogeny of craniofacial development in sympatric Arctic charr morphs. Developmental Dynamics 244, 1168–1178. 26150089 10.1002/dvdy.24302

[brv12534-bib-0129] Kapralova, K. H. , Morrissey, M. B. , Kristjánsson, B. K. , Ólafsdóttir, G. A. , Snorrason, S. S. & Ferguson, M. M. (2011). Evolution of adaptive diversity and genetic connectivity in Arctic charr (*Salvelinus alpinus*) in Iceland. Heredity 106, 472–487. 21224880 10.1038/hdy.2010.161 PMC3131972

[brv12534-bib-0130] Karvonen, A. , Lundsgaard‐Hansen, B. , Jokela, J. & Seehausen, O. (2013). Differentiation in parasitism among ecotypes of whitefish segregating along depth gradients. Oikos 122, 122–128.

[brv12534-bib-0131] Keller, I. & Seehausen, O. (2012). Thermal adaptation and ecological speciation. Molecular Ecology 21, 782–799. 22182048 10.1111/j.1365-294X.2011.05397.x

[brv12534-bib-0132] Kingsolver, J. G. , Hoekstra, H. E. , Hoekstra, J. M. , Berrigan, D. , Vignieri, S. N. , Hill, C. E. , Hoang, A. , Gibert, P. & Beerli, P. (2001). The strength of phenotypic selection in natural populations. American Naturalist 157, 245–261. 10.1086/319193 18707288

[brv12534-bib-0133] Kinnison, M. T. , Hairston, N. G. & Hendry, A. P. (2015). Cryptic eco‐evolutionary dynamics In Year in Evolutionary Biology *Annals of the New York Academy of Sciences* (Volume 1360, eds Mousseau T. A. and Fox C. W.), pp. 120–144. Blackwell Science Publication, Oxford. 10.1111/nyas.12974 26619300

[brv12534-bib-0134] Kirschner, M. & Gerhart, J. (1998). Evolvability. Proceedings of the National Academy of Sciences of the United States of America 95, 8420–8427. 9671692 10.1073/pnas.95.15.8420 PMC33871

[brv12534-bib-0135] Kitano, J. , Kawagishi, Y. , Mori, S. , Peichel, C. L. , Makino, T. , Kawata, M. & Kusakabe, M. (2011). Divergence in sex steroid hormone signaling between sympatric species of Japanese threespine stickleback. PLoS One 6, e29253. 10.1371/journal.pone.0029253 PMC3247238 22216225

[brv12534-bib-0136] * Klemetsen, A. (2010). The charr problem revisited: exceptional phenotypic plasticity promotes cological speciation in postglacial lakes. Freshwater Reviews 3, 49–74.

[brv12534-bib-0137] Klemola, T. , Tanhuanpaa, M. , Korpimaki, E. & Ruohomaki, K. (2002). Specialist and generalist natural enemies as an explanation for geographical gradients in population cycles of northern herbivores. Oikos 99, 83–94.

[brv12534-bib-0138] Knudsen, R. , Amundsen, P. A. & Klemetsen, A. (2003). Inter‐ and intra‐morph patterns in helminth communities of sympatric whitefish morphs. Journal of Fish Biology 62, 847–859.

[brv12534-bib-0139] Knudsen, R. , Primicerio, R. , Amundsen, P. A. & Klemetsen, A. (2010). Temporal stability of individual feeding specialization may promote speciation. Journal of Animal Ecology 79, 161–168. 19796292 10.1111/j.1365-2656.2009.01625.x

[brv12534-bib-0140] Knudsen, R. , Siwertsson, A. , Adams, C. E. , Garduno‐Paz, M. , Newton, J. & Amundsen, P. A. (2011). Temporal stability of niche use exposes sympatric Arctic charr to alternative selection pressures. Evolutionary Ecology 25, 589–604.

[brv12534-bib-0141] Kristjánsson, B. K. , Leblanc, C. A.‐L. , Skúlason, S. , Snorrason, S. S. & Noakes, D. L. G. (2018). Phenotypic plasticity in the morphology of small benthic Icelandic Arctic charr (*Salvelinus alpinus*). Ecology of Freshwater Fish 27, 636–645.

[brv12534-bib-0142] * Kristjánsson, B. K. , Skúlason, S. & Noakes, D. L. G. (2002a). Morphological segregation of Icelandic threespine stickleback (*Gasterosteus aculeatus* L). Biological Journal of the Linnean Society 76, 247–257.

[brv12534-bib-0143] Kristjánsson, B. K. , Skúlason, S. & Noakes, D. L. G. (2002b). Rapid divergence in a recently isolated population of threespine stickleback (*Gasterosteus aculeatus* L.). Evolutionary Ecology Research 4, 659–672.

[brv12534-bib-0144] Kristjánsson, B. K. , Skúlason, S. , Snorrason, S. S. & Noakes, D. L. G. (2012). Fine‐scale parallel patterns in diversity of small benthic Arctic charr (*Salvelinus alpinus*) in relation to the ecology of lava/groundwater habitats. Ecology and Evolution 2, 1099–1112. 22833787 10.1002/ece3.235 PMC3402187

[brv12534-bib-0145] Küttner, E. , Parsons, K. J. , Easton, A. A. , Skúlason, S. , Danzmann, R. G. & Ferguson, M. M. (2014). Hidden genetic variation evolves with ecological specialization: the genetic basis of phenotypic plasticity in Arctic charr ecomorphs. Evolution & Development 16, 247–257. 24920458 10.1111/ede.12087

[brv12534-bib-0146] Küttner, E. , Parsons, K. J. , Robinson, B. W. , Skúlason, S. , Danzmann, R. G. & Ferguson, M. M. (2013). Effects of population, family, and diet on craniofacial morphology of Icelandic Arctic charr (*Salvelinus alpinus*). Biological Journal of the Linnean Society 108, 702–714.

[brv12534-bib-0147] Laland, K. , Matthews, B. & Feldman, M. W. (2016). An introduction to niche construction theory. Evolutionary Ecology 30, 191–202. 27429507 10.1007/s10682-016-9821-z PMC4922671

[brv12534-bib-0148] Laland, K. N. , Uller, T. , Fellman, M. W. , Sterelny, K. , Muller, G. B. , Moczek, A. , Jablonka, E. & Odling‐Smee, J. (2015). The extended evolutionary synthesis: its structure, assumptions and predictions. Proceedings of the Royal Society B‐Biological Sciences 282, 14. 10.1098/rspb.2015.1019 PMC4632619 26246559

[brv12534-bib-0149] Laporte, M. , Rogers, S. M. , Dion‐Cote, A. M. , Normandeau, E. & Gagnaire, P. A. (2015). RAD‐QTL mapping reveals both genome‐level parallelism and different genetic architecture underlying the evolution of body shape in lake whitefish (*Coregonus clupeaformis*) species pairs. G3‐Genes Genomes Genetics 5, 2919–2919. 10.1534/g3.115.019067 PMC4502382 26002924

[brv12534-bib-0150] Le Luyer, J. , Laporte, M. , Beacham, T. D. , Kaukinen, K. H. , Withler, R. E. , Leong, J. S. , Rondeau, E. B. , Koop, B. F. & Bernatchez, L. (2017). Parallel epigenetic modifications induced by hatchery rearing in a Pacific salmon. Proceedings of the National Academy of Sciences 114, 12964–12969. 10.1073/pnas.1711229114 PMC5724268 29162695

[brv12534-bib-0151] Leblanc, C. A. L. , Benhaim, D. , Hansen, B. R. , Kristjánsson, B. K. & Skúlason, S. (2011). The importance of egg size and social effects for behaviour of Arctic charr juveniles. Ethology 117, 664–674.

[brv12534-bib-0152] Ledon‐Rettig, C. C. & Pfennig, D. W. (2011). Emerging model systems in eco‐evo‐devo: the environmentally responsive spadefoot toad. Evolution & Development 13, 391–400. 21740512 10.1111/j.1525-142X.2011.00494.x

[brv12534-bib-0153] Levis, N. A. , Isdaner, A. J. & Pfennig, D. W. (2018). Morphological novelty emerges from pre‐existing phenotypic plasticity. Nature Ecology & Evolution 2, 1289–1297. 29988161 10.1038/s41559-018-0601-8

[brv12534-bib-0154] Lien, S. , Koop, B. F. , Sandve, S. R. , Miller, J. R. , Kent, M. P. , Nome, T. , Hvidsten, T. R. , Leong, J. S. , Minkley, D. R. , Zimin, A. , Grammes, F. , Grove, H. , Gjuvsland, A. , Walenz, B. , Hermansen, R. A. , et al. (2016). The Atlantic salmon genome provides insights into rediploidization. Nature 533, 200–205. 27088604 10.1038/nature17164 PMC8127823

[brv12534-bib-0155] Ljunggren, L. & Sandstrom, A. (2007). Influence of visual conditions on foraging and growth of juvenile fishes with dissimilar sensory physiology. Journal of Fish Biology 70, 1319–1334.

[brv12534-bib-0156] Loh, Y. H. E. , Katz, L. S. , Mims, M. C. , Kocher, T. D. , Yi, S. V. & Streelman, J. T. (2008). Comparative analysis reveals signatures of differentiation amid genomic polymorphism in Lake Malawi cichlids. Genome Biology 9, R113. 18616806 10.1186/gb-2008-9-7-r113 PMC2530870

[brv12534-bib-0157] Loreau, M. , Naeem, S. , Inchausti, P. , Bengtsson, J. , Grime, J. P. , Hector, A. , Hooper, D. U. , Huston, M. A. , Raffaelli, D. , Schmid, B. , Tilman, D. & Wardle, D. A. (2001). Ecology ‐ biodiversity and ecosystem functioning: current knowledge and future challenges. Science 294, 804–808. 11679658 10.1126/science.1064088

[brv12534-bib-0158] Lu, G. Q. & Bernatchez, L. (1999). Correlated trophic specialization and genetic divergence in sympatric lake whitefish ecotypes (*Coregonus clupeaformis*): support for the ecological speciation hypothesis. Evolution 53, 1491–1505. 28565561 10.1111/j.1558-5646.1999.tb05413.x

[brv12534-bib-0159] Lundsgaard‐Hansen, B. , Matthews, B. & Seehausen, O. (2014). Ecological speciation and phenotypic plasticity affect ecosystems. Ecology 95, 2723–2735.

[brv12534-bib-0160] Lundsgaard‐Hansen, B. , Matthews, B. , Vonlanthen, P. , Taverna, A. & Seehausen, O. (2013). Adaptive plasticity and genetic divergence in feeding efficiency during parallel adaptive radiation of whitefish (*Coregonus* spp.). Journal of Evolutionary Biology 26, 483–498. 23286233 10.1111/jeb.12063

[brv12534-bib-0161] MacColl, A. D. C. (2011). The ecological causes of evolution. Trends in Ecology & Evolution 26, 514–522. 21763030 10.1016/j.tree.2011.06.009

[brv12534-bib-0162] Macqueen, D. J. , Kristjánsson, B. K. , Paxton, C. G. M. , Vieira, V. L. A. & Johnston, I. A. (2011). The parallel evolution of dwarfism in Arctic charr is accompanied by adaptive divergence in mTOR‐pathway gene expression. Molecular Ecology 20, 3167–3184. 21714822 10.1111/j.1365-294X.2011.05172.x

[brv12534-bib-0163] Malinen, T. , Tuomaala, A. , Lehtonen, H. & Kahilainen, K. K. (2014). Hydroacoustic assessment of mono‐ and polymorphic *Coregonus* density and biomass in subarctic lakes. Ecology of Freshwater Fish 23, 424–437.

[brv12534-bib-0164] Matthews, B. , Aebischer, T. , Sullam, K. E. , Lundsgaard‐Hansen, B. & Seehausen, O. (2016). Experimental evidence of an eco‐evolutionary feedback during adaptive divergence. Current Biology 26, 483–489. 26804555 10.1016/j.cub.2015.11.070

[brv12534-bib-0165] Matthews, B. , Best, R. J. , Feulner, P. G. D. , Narwani, A. & Limberger, R. (2018). Evolution as an ecosystem process: insights from genomics. Genome 61, 298–309. 29241022 10.1139/gen-2017-0044

[brv12534-bib-0166] Matthews, B. , De Meester, L. , Jones, C. G. , Ibelings, B. W. , Bouma, T. J. , Nuutinen, V. , van de Koppel, J. & Odling‐Smee, J. (2014). Under niche construction: an operational bridge between ecology, evolution, and ecosystem science. Ecological Monographs 84, 245–263.

[brv12534-bib-0167] Matthews, B. , Narwani, A. , Hausch, S. , Nonaka, E. , Peter, H. , Yamamichi, M. , Sullam, K. E. , Bird, K. C. , Thomas, M. K. , Hanley, T. C. & Turner, C. B. (2011). Toward an integration of evolutionary biology and ecosystem science. Ecology Letters 14, 690–701. 21554512 10.1111/j.1461-0248.2011.01627.x

[brv12534-bib-0168] McCairns, R. J. S. & Bernatchez, L. (2010). Adaptive divergence between freshwater and marine sticklebacks: insight into the role of phenotypic plasticity from an integrated analysis of candidate gene expression. Evolution 64, 1029–1047. 19895556 10.1111/j.1558-5646.2009.00886.x

[brv12534-bib-0169] * McCairns, R. J. S. & Fox, M. G. (2004). Habitat and home range fidelity in a trophically dimorphic pumpkinseed sunfish (*Lepomis gibbosus*) population. Oecologia 140, 271–279. 16228261 10.1007/s00442-004-1580-9

[brv12534-bib-0170] * Mccart, P. (1970). Evidence for the existence of sibling species of pygmy whitefish (*Prosopium coulteri*) in three Alaskan lakes In Biology of Coregonid Fishes (eds Lindsey C. C. and Woods C. S.), pp. 81–98. University of Manitoba Press, Winnipeg.

[brv12534-bib-0171] McKay, B. D. & Zink, R. M. (2015). Sisyphean evolution in Darwin's finches. Biological Reviews 90, 689–698. 25040800 10.1111/brv.12127

[brv12534-bib-0172] * McPhail, J. D. (1984). Ecology and evolution of sympatric sticklebacks (*Gasterosteus*): morphological and genetic evidence for a species pair in Enos Lake, British Columbia. Canadian Journal of Zoology 62, 1402–1408.

[brv12534-bib-0173] Metz, J. A. J. , Nisbet, R. M. & Geritz, S. A. H. (1992). How should we define fitness for general ecological scenarios. Trends in Ecology & Evolution 7, 198–202. 21236007 10.1016/0169-5347(92)90073-K

[brv12534-bib-0174] Minelli, A. (2015). Grand challenges in evolutionary developmental biology. Frontiers in Ecology and Evolution 2, 85.

[brv12534-bib-0175] Miner, B. G. , Sultan, S. E. , Morgan, S. G. , Padilla, D. K. & Relyea, R. A. (2005). Ecological consequences of phenotypic plasticity. Trends in Ecology & Evolution 20, 685–692. 16701458 10.1016/j.tree.2005.08.002

[brv12534-bib-0176] Mittelbach, G. G. , Turner, A. M. , Hall, D. J. , Rettig, J. E. & Osenberg, C. W. (1995). Perturbation and resilience ‐ a long‐term, whole‐lake study of predator extinction and reintroduction. Ecology 76, 2347–2360.

[brv12534-bib-0177] Moczek, A. P. (2012). The nature of nurture and the future of evodevo: toward a theory of developmental evolution. Integrative and Comparative Biology 52, 108–119. 22617162 10.1093/icb/ics048

[brv12534-bib-0178] Moczek, A. P. (2015). Re‐evaluating the environment in developmental evolution. Frontiers in Ecology and Evolution 3, 7.

[brv12534-bib-0179] Moczek, A. P. , Sears, K. E. , Stollewerk, A. , Wittkopp, P. J. , Diggle, P. , Dworkin, I. , Ledon‐Rettig, C. , Matus, D. Q. , Roth, S. , Abouheif, E. , Brown, F. D. , Chiu, C. H. , Cohen, C. S. , De Tomaso, A. W. , Gilbert, S. F. , et al. (2015). The significance and scope of evolutionary developmental biology: a vision for the 21st century. Evolution & Development 17, 198–219. 25963198 10.1111/ede.12125

[brv12534-bib-0180] Moczek, A. P. , Sultan, S. , Foster, S. , Ledon‐Rettig, C. , Dworkin, I. , Nijhout, H. F. , Abouheif, E. & Pfennig, D. W. (2011). The role of developmental plasticity in evolutionary innovation. Proceedings of the Royal Society B‐Biological Sciences 278, 2705–2713. 10.1098/rspb.2011.0971 PMC3145196 21676977

[brv12534-bib-0181] * Moore, S. A. & Bronte, C. R. (2001). Delineation of sympatric morphotypes of lake trout in Lake Superior. Transactions of the American Fisheries Society 130, 1233–1240.

[brv12534-bib-0182] Morris, M. R. J. , Richard, R. , Leder, E. H. , Barrett, R. D. H. , Aubin‐Horth, N. & Rogers, S. M. (2014). Gene expression plasticity evolves in response to colonization of freshwater lakes in threespine stickleback. Molecular Ecology 23, 3226–3240. 24889067 10.1111/mec.12820

[brv12534-bib-0183] Mousseau, T. A. & Fox, C. W. (1998). The adaptive significance of maternal effects. Trends in Ecology & Evolution 13, 403–407. 21238360 10.1016/s0169-5347(98)01472-4

[brv12534-bib-0184] Nonaka, E. , Svanbäck, R. , Thibert‐Plante, X. , Englund, G. & Brännström, Å. (2015). Mechanisms by which phenotypic plasticity affects adaptive divergence and ecological speciation. American Naturalist 186, E126–E143. 10.1086/683231 26655782

[brv12534-bib-0185] Nordeng, H. (1983). Solution to the "charr problem" based on Arctic char (*Salvelinus alpinus*) in Norway. Canadian Journal of Fisheries and Aquatic Sciences 40, 1372–1387.

[brv12534-bib-0186] Nosil, P. (2012). Ecological Speciation. Oxford University Press, Oxford.

[brv12534-bib-0187] Odling‐Smee, J. , Erwin, D. H. , Palkovacs, E. P. , Feldman, M. W. & Laland, K. N. (2013). Niche construction theory: a practical guide for ecologists. Quarterly Review of Biology 88, 3–28. 10.1086/669266 23653966

[brv12534-bib-0188] * Ohlberger, J. , Mehner, T. , Staaks, G. & Holker, F. (2008). Temperature‐related physiological adaptations promote ecological divergence in a sympatric species pair of temperate freshwater fish, *Coregonus* spp. Functional Ecology 22, 501–508.

[brv12534-bib-0189] Oke, K. B. , Bukhari, M. , Kaeuffer, R. , Rolshausen, G. , Rasanen, K. , Bolnick, D. I. , Peichel, C. L. & Hendry, A. P. (2016). Does plasticity enhance or dampen phenotypic parallelism? A test with three lake‐stream stickleback pairs. Journal of Evolutionary Biology 29, 126–143. 26411538 10.1111/jeb.12767

[brv12534-bib-0190] * Ólafsdóttir, G. A. , Ritchie, M. G. & Snorrason, S. S. (2006). Positive assortative mating between recently described sympatric morphs of Icelandic sticklebacks. Biology Letters 2, 250–252. 17148375 10.1098/rsbl.2006.0456 PMC1618888

[brv12534-bib-0191] * Ólafsdóttir, G. A. , Snorrason, S. S. & Ritchie, M. G. (2007a). Morphological and genetic divergence of intralacustrine stickleback morphs in Iceland: a case for selective differentiation? Journal of Evolutionary Biology 20, 603–616. 17305827 10.1111/j.1420-9101.2006.01250.x

[brv12534-bib-0192] * Ólafsdóttir, G. A. , Snorrason, S. S. & Ritchie, M. G. (2007b). Postglacial intra‐lacustrine divergence of Icelandic threespine stickleback morphs in three neovolcanic lakes. Journal of Evolutionary Biology 20, 1870–1881. 17714304 10.1111/j.1420-9101.2007.01375.x

[brv12534-bib-0193] * Olsson, J. , Svanbäck, R. & Eklöv, P. (2006). Growth rate constrain morphological divergence when driven by competition. Oikos 115, 15–22.

[brv12534-bib-0194] * Olsson, J. , Svanbäck, R. & Eklöv, P. (2007). Effects of resource level and habitat type on behavioral and morphological plasticity in Eurasian perch. Oecologia 152, 48–56. 17431684 10.1007/s00442-006-0588-8

[brv12534-bib-0195] * Østbye, K. , Amundsen, P. A. , Bernatchez, L. , Klemetsen, A. , Knudsen, R. , Kristoffersen, R. , Næsje, T. F. & Hindar, K. (2006). Parallel evolution of ecomorphological traits in the European whitefish *Coregonus lavaretus* (L.) species complex during postglacial times. Molecular Ecology 15, 3983–4001. 17054498 10.1111/j.1365-294X.2006.03062.x

[brv12534-bib-0196] * Østbye, K. , Næsje, T. F. , Bernatchez, L. , Sandlund, O. T. & Hindar, K. (2005). Morphological divergence and origin of sympatric populations of European whitefish (*Coregonus lavaretus* L.) in Lake Femund, Norway. Journal of Evolutionary Biology 18, 683–702. 15842498 10.1111/j.1420-9101.2004.00844.x

[brv12534-bib-0197] * Ozerov, M. Y. , Himberg, M. , Debes, P. V. , Hägerstrand, H. & Vasemägi, A. (2016). Combining genetic markers with an adaptive meristic trait improves performance of mixed‐stock analysis in Baltic whitefish. Ices Journal of Marine Science 73, 2529–2538.

[brv12534-bib-0198] Palkovacs, E. P. & Post, D. M. (2009). Experimental evidence that phenotypic divergence in predators drives community divergence in prey. Ecology 90, 300–305. 19323211 10.1890/08-1673.1

[brv12534-bib-0199] Park, B. K. , Lee, Y. S. & Park, S. S. (2007). Calculation of search volume on cruise‐searching planktivorous fish in foraging model. Journal of Environmental Biology 28, 537–543. 18380072

[brv12534-bib-0200] Parsons, K. J. & Albertson, R. C. (2013). Unifying and generalizing the two strands of evo‐devo. Trends in Ecology & Evolution 28, 584–591. 23876674 10.1016/j.tree.2013.06.009

[brv12534-bib-0201] Parsons, K. J. & Robinson, B. W. (2006). Replicated evolution of integrated plastic responses during early adaptive divergence. Evolution 60, 801–813. 16739461

[brv12534-bib-0202] Parsons, K. J. & Robinson, B. W. (2007). Foraging performance of diet‐induced morphotypes in pumpkinseed sunfish (*Lepomis gibbosus*) favours resource polymorphism. Journal of Evolutionary Biology 20, 673–684. 17305833 10.1111/j.1420-9101.2006.01249.x

[brv12534-bib-0203] Parsons, K. J. , Concannon, M. , Navon, D. , Wang, J. , Ea, I. , Groveas, K. , Campbell, C. & Albertson, R. C. (2016). Foraging environment determines the genetic architecture and evolutionary potential of trophic morphology in cichlid fishes. Molecular Ecology 25, 6012–6023. 27516345 10.1111/mec.13801

[brv12534-bib-0204] Parsons, K. J. , Sheets, H. D. , Skúlason, S. & Ferguson, M. M. (2011). Phenotypic plasticity, heterochrony and ontogenetic repatterning during juvenile development of divergent Arctic charr (*Salvelinus alpinus*). Journal of Evolutionary Biology 24, 1640–1652. 21599773 10.1111/j.1420-9101.2011.02301.x

[brv12534-bib-0205] Parsons, K. J. , Skúlason, S. & Ferguson, M. (2010). Morphological variation over ontogeny and environments in resource polymorphic arctic charr (*Salvelinus alpinus*). Evolution & Development 12, 246–257. 20565535 10.1111/j.1525-142X.2010.00410.x

[brv12534-bib-0206] Parsons, K. J. , Trent Taylor, A. , Powder, K. E. & Albertson, R. C. (2014). Wnt signalling underlies the evolution of new phenotypes and craniofacial variability in Lake Malawi cichlids. Nature Communications 5, 3629. 10.1038/ncomms4629 PMC4238940 24699776

[brv12534-bib-0207] * Pavey, S. A. , Sevellec, M. , Adam, W. , Normandeau, E. , Lamaze, F. C. , Gagnaire, P. A. , Filteau, M. , Hebert, F. O. , Maaroufi, H. & Bernatchez, L. (2013). Nonparallelism in MHCII diversity accompanies nonparallelism in pathogen infection of lake whitefish (*Coregonus clupeaformis*) species pairs as revealed by next‐generation sequencing. Molecular Ecology 22, 3833–3849. 23786238 10.1111/mec.12358

[brv12534-bib-0208] Peichel, C. L. (2005). Fishing for the secrets of vertebrate evolution in threespine sticklebacks. Developmental Dynamics 234, 815–823. 16252286 10.1002/dvdy.20564

[brv12534-bib-0209] Peichel, C. L. , Sullivan, S. T. , Liachko, I. & White, M. A. (2017). Improvement of the threespine stickleback genome using a Hi‐C‐based proximity‐guided assembly. Journal of Heredity 108, 693–700. 28821183 10.1093/jhered/esx058 PMC5892396

[brv12534-bib-0210] * Pelletier, F. , Garant, D. & Hendry, A. P. (2009). Eco‐evolutionary dynamics. Philosophical Transactions of the Royal Society B‐Biological Sciences 364, 1483–1489. 10.1098/rstb.2009.0027 PMC2690510 19414463

[brv12534-bib-0211] Penney, H. , Beirão, J. & Purchase, C. (2018). Phenotypic plasticity during external embryonic development is affected more by maternal effects than multiple abiotic factors in brook trout. Evolutionary Ecology Research 19, 171–194.

[brv12534-bib-0212] * Perreault‐Payette, A. , Muir, A. M. , Goetz, F. , Perrier, C. , Normandeau, E. , Sirois, P. & Bernatchez, L. (2017). Investigating the extent of parallelism in morphological and genomic divergence among lake trout ecotypes in Lake Superior. Molecular Ecology 26, 1477–1497. 28099784 10.1111/mec.14018

[brv12534-bib-0213] Perrier, C. , Bourret, V. , Kent, M. P. & Bernatchez, L. (2013). Parallel and nonparallel genome‐wide divergence among replicate population pairs of freshwater and anadromous Atlantic salmon. Molecular Ecology 22, 5577–5593. 24730037 10.1111/mec.12500

[brv12534-bib-0214] Persson, L. (1986). Effects of reduced interspecific competition on resource utilization in perch (*Perca fluviatilis*). Ecology 67, 355–364.

[brv12534-bib-0215] Persson, L. , De Roos, A. M. , Claessen, D. , Byström, P. , Lövgren, J. , Sjögren, S. , Svanbäck, R. , Wahlström, E. & Westman, E. (2003). Gigantic cannibals driving a whole‐lake trophic cascade. Proceedings of the National Academy of Sciences of the United States of America 100, 4035–4039. 12646706 10.1073/pnas.0636404100 PMC153043

[brv12534-bib-0216] Pfennig, D. (2016). Ecological evolutionary developmental biology In Encyclopedia of Evolutionary Biology (Volume 1, ed. Kliman R. M.), pp. 474–481. Academic Press, Oxford.

[brv12534-bib-0217] Pfennig, D. W. & Pfennig, K. S. (2012). Evolution's Wedge: Competition and the Origins of Diversity. University of California Press, Berkely.

[brv12534-bib-0218] Pfennig, D. W. , Wund, M. A. , Snell‐Rood, E. C. , Cruickshank, T. , Schlichting, C. D. & Moczek, A. P. (2010). Phenotypic plasticity's impacts on diversification and speciation. Trends in Ecology & Evolution 25, 459–467. 20557976 10.1016/j.tree.2010.05.006

[brv12534-bib-0219] Pigliucci, M. (2008). Opinion ‐ is evolvability evolvable? Nature Reviews Genetics 9, 75–82. 10.1038/nrg2278 18059367

[brv12534-bib-0220] Plaistow, S. J. , Lapsley, C. T. & Benton, T. G. (2006). Context‐dependent intergenerational effects: the interaction between past and present environments and its effect on population dynamics. American Naturalist 167, 206–215. 10.1086/499380 16670981

[brv12534-bib-0221] Post, D. M. & Palkovacs, E. P. (2009). Eco‐evolutionary feedbacks in community and ecosystem ecology: interactions between the ecological theatre and the evolutionary play. Philosophical Transactions of the Royal Society B‐Biological Sciences 364, 1629–1640. 10.1098/rstb.2009.0012 PMC2690506 19414476

[brv12534-bib-0222] * Præbel, K. , Knudsen, R. , Siwertsson, A. , Karhunen, M. , Kahilainen, K. K. , Ovaskainen, O. , Østbye, K. , Peruzzi, S. , Fevolden, S. E. & Amundsen, P. A. (2013). Ecological speciation in postglacial European whitefish: rapid adaptive radiations into the littoral, pelagic, and profundal lake habitats. Ecology and Evolution 3, 4970–4986. 24455129 10.1002/ece3.867 PMC3892361

[brv12534-bib-0223] * Proulx, R. & Magnan, P. (2004). Contribution of phenotypic plasticity and heredity to the trophic polymorphism of lacustrine brook charr (*Salvelinus fontinalis* M.). Evolutionary Ecology Research 6, 503–522.

[brv12534-bib-0224] * Quevedo, M. , Svanbäck, R. & Eklöv, P. (2009). Intrapopulation niche partitioning in a generalist predator limits food web connectivity. Ecology 90, 2263–2274. 19739388 10.1890/07-1580.1

[brv12534-bib-0225] Raffard, A. , Santoul, F. , Cucherousset, J. & Blanchet, S. (2019). The community and ecosystem consequences of intraspecific diversity: a meta‐analysis. Biological Reviews 94, 648–661. 30294844 10.1111/brv.12472

[brv12534-bib-0226] Ran, F. A. , Hsu, P. D. , Wright, J. , Agarwala, V. , Scott, D. A. & Zhang, F. (2013). Genome engineering using the CRISPR‐Cas9 system. Nature Protocols 8, 2281–2308. 24157548 10.1038/nprot.2013.143 PMC3969860

[brv12534-bib-0227] * Räsänen, K. & Hendry, A. P. (2008). Disentangling interactions between adaptive divergence and gene flow when ecology drives diversification. Ecology Letters 11, 624–636. 18384363 10.1111/j.1461-0248.2008.01176.x

[brv12534-bib-0228] Räsänen, K. & Kruuk, L. E. B. (2007). Maternal effects and evolution at ecological time‐scales. Functional Ecology 21, 408–421.

[brv12534-bib-0229] Rennison, D. J. , Heilbron, K. , Barrett, R. D. H. & Schluter, D. (2015). Discriminating selection on lateral plate phenotype and its underlying gene, Ectodysplasin, in Threespine stickleback. American Naturalist 185, 150–156. 10.1086/679280 25560560

[brv12534-bib-0230] Robinson, B. W. & Parsons, K. J. (2002). Changing times, spaces, and faces: tests and implications of adaptive morphological plasticity in the fishes of northern postglacial lakes. Canadian Journal of Fisheries and Aquatic Sciences 59, 1819–1833.

[brv12534-bib-0231] Robinson, B. W. & Schluter, D. (2000). Natural selection and the evolution of adaptive genetic variation in northern freshwater fishes In Adaptive Genetic Variation in the Wild (eds Mousseau T. A., Sinervo B. and Endler J. A.), pp. 65–94. Oxford University Press, New York.

[brv12534-bib-0232] Robinson, B. W. & Wilson, D. S. (1994). Character release and displacement in fishes ‐ a neglected literature. American Naturalist 144, 596–627.

[brv12534-bib-0233] Robinson, B. W. & Wilson, D. S. (1996). Genetic variation and phenotypic plasticity in a trophically polymorphic population of pumpkinseed sunfish (*Lepomis gibbosus*). Evolutionary Ecology 10, 631–652.

[brv12534-bib-0234] * Robinson, B. W. , Wilson, D. S. & Margosian, A. S. (2000). A pluralistic analysis of character release in pumpkinseed sunfish (*Lepomis gibbosus*). Ecology 81, 2799–2812.

[brv12534-bib-0235] * Robinson, B. W. , Wilson, D. S. , Margosian, A. S. & Lotito, P. T. (1993). Ecological and morphological‐differentiation of pumpkinseed sunfish in lakes without bluegill sunfish. Evolutionary Ecology 7, 451–464.

[brv12534-bib-0236] * Robinson, B. W. , Wilson, D. S. & Shea, G. O. (1996). Trade‐offs of ecological specialization: an intraspecific comparison of pumpkinseed sunfish phenotypes. Ecology 77, 170–178.

[brv12534-bib-0237] Roesti, M. , Gavrilets, S. , Hendry, A. P. , Salzburger, W. & Berner, D. (2014). The genomic signature of parallel adaptation from shared genetic variation. Molecular Ecology 23, 3944–3956. 24635356 10.1111/mec.12720 PMC4122612

[brv12534-bib-0238] * Rogers, S. M. & Bernatchez, L. (2006). The genetic basis of intrinsic and extrinsic post‐zygotic reproductive isolation jointly promoting speciation in the lake whitefish species complex (*Coregonus clupeaformis*). Journal of Evolutionary Biology 19, 1979–1994. 17040396 10.1111/j.1420-9101.2006.01150.x

[brv12534-bib-0239] Rohner, N. , Jarosz, D. F. , Kowalko, J. E. , Yoshizawa, M. , Jeffery, W. R. , Borowsky, R. L. , Lindquist, S. & Tabin, C. J. (2013). Cryptic variation in morphological evolution: HSP90 as a capacitor for loss of eyes in cavefish. Science 342, 1372–1375. 24337296 10.1126/science.1240276 PMC4004346

[brv12534-bib-0240] Rooney, N. & McCann, K. S. (2012). Integrating food web diversity, structure and stability. Trends in Ecology & Evolution 27, 40–46. 21944861 10.1016/j.tree.2011.09.001

[brv12534-bib-0241] Rudman, S. M. , Barbour, M. A. , Csilléry, K. , Gienapp, P. , Guillaume, F. , Hairston, N. G. Jr. , Hendry, A. P. , Lasky, J. R. , Rafajlović, M. , Räsänen, K. , Schmidt, P. S. , Seehausen, O. , Therkildsen, N. O. , Turcotte, M. M. & Levine, J. M. (2017). What genomic data can reveal about eco‐evolutionary dynamics. Nature Ecology & Evolution 2, 9–15. 29158555 10.1038/s41559-017-0385-2

[brv12534-bib-0242] Sæther, B. E. & Engen, S. (2015). The concept of fitness in fluctuating environments. Trends in Ecology & Evolution 30, 273–281. 25843273 10.1016/j.tree.2015.03.007

[brv12534-bib-0243] * Saint‐Laurent, R. , Legault, M. & Bernatchez, L. (2003). Divergent selection maintains adaptive differentiation despite high gene flow between sympatric rainbow smelt ecotypes (*Osmerus mordax* Mitchill). Molecular Ecology 12, 315–330. 12535084 10.1046/j.1365-294x.2003.01735.x

[brv12534-bib-0244] Saltz, J. B. & Nuzhdin, S. V. (2014). Genetic variation in niche construction: implications for development and evolutionary genetics. Trends in Ecology & Evolution 29, 8–14. 24126050 10.1016/j.tree.2013.09.011 PMC3874263

[brv12534-bib-0245] Sanderson, B. L. , Hrabik, T. R. , Magnuson, J. J. & Post, D. M. (1999). Cyclic dynamics of a yellow perch (*Perca flavescens*) population in an oligotrophic lake: evidence for the role of intraspecific interactions. Canadian Journal of Fisheries and Aquatic Sciences 56, 1534–1542.

[brv12534-bib-0246] * Sandlund, O. T. , Gunnarsson, K. , Jónasson, P. M. , Jonsson, B. , Lindem, T. , Magnússon, K. P. , Malmquist, H. J. , Sigurjónsdóttir, H. , Skúlason, S. & Snorrason, S. S. (1992). The Arctic Charr *Salvelinus alpinus* in Thingvallavatn. Oikos 64, 305–351.

[brv12534-bib-0247] Santos, M. E. , Berger, C. S. , Refki, P. N. & Khila, A. (2015). Integrating evo‐devo with ecology for a better understanding of phenotypic evolution. Briefings in Functional Genomics 14, 384–395. 25750411 10.1093/bfgp/elv003 PMC4652033

[brv12534-bib-0248] Scheiner, S. M. (1993). Genetics and evolution of phenotypic plasticity. Annual Review of Ecology and Systematics 24, 35–68.

[brv12534-bib-0249] Schlichting, C. D. & Wund, M. A. (2014). Phenotypic plasticity and epigenetic marking: an assessment of evidence for genetic accomodation. Evolution 68, 656–672. 24410266 10.1111/evo.12348

[brv12534-bib-0250] * Schluter, D. (1993). Adaptive radiation in Sticklebacks ‐ size, shape, and habitat use efficiency. Ecology 74, 699–709.

[brv12534-bib-0251] * Schluter, D. (1995). Adaptive radiation in sticklebacks ‐ trade‐offs in feeding performance and growth. Ecology 76, 82–90.

[brv12534-bib-0252] Schluter, D. (2000). The Ecology of Adaptive Radiation. Oxford University Press, Oxford.

[brv12534-bib-0253] * Schluter, D. & Mcphail, J. D. (1992). Ecological character displacement and speciation in sticklebacks. American Naturalist 140, 85–108. 10.1086/285404 19426066

[brv12534-bib-0254] Schmitz, O. J. (2006). Predators have large effects on ecosystem properties by changing plant diversity, not plant biomass. Ecology 87, 1432–1437. 16869417 10.1890/0012-9658(2006)87[1432:phleoe]2.0.co;2

[brv12534-bib-0255] Schneider, R. F. & Meyer, A. (2017). How plasticity, genetic assimilation and cryptic genetic variation may contribute to adaptive radiations. Molecular Ecology 26, 330–350. 27747962 10.1111/mec.13880

[brv12534-bib-0256] Schoener, T. W. (2011). The newest synthesis: understanding the interplay of evolutionary and ecological dynamics. Science 331, 426–429. 21273479 10.1126/science.1193954

[brv12534-bib-0257] * Schulz, M. , Freyhof, J. , Saint‐Laurent, R. , Østbye, K. , Mehner, T. & Bernatchez, L. (2006). Evidence for independent origin of two spring‐spawning ciscoes (Salmoniformes: Coregonidae) in Germany. Journal of Fish Biology 68, 119–135.

[brv12534-bib-0258] Schwab, D. B. , Casasa, S. & Moczek, A. P. (2019). On the reciprocally causal and constructive nature of developmental plasticity and robustness. Frontiers in Genetics 9, 735. 30687394 10.3389/fgene.2018.00735 PMC6335315

[brv12534-bib-0259] * Seehausen, O. & Wagner, C. E. (2014). Speciation in freshwater fishes. Annual Review of Ecology, Evolution, and Systematics 45, 621–651.

[brv12534-bib-0260] * Segers, F. , Berishvili, G. & Taborsky, B. (2012). Egg size‐dependent expression of growth hormone receptor accompanies compensatory growth in fish. Proceedings of the Royal Society B‐Biological Sciences 279, 592–600. 10.1098/rspb.2011.1104 PMC3234566 21752823

[brv12534-bib-0261] Shama, L. N. S. & Wegner, K. M. (2014). Grandparental effects in marine sticklebacks: transgenerational plasticity across multiple generations. Journal of Evolutionary Biology 27, 2297–2307. 25264208 10.1111/jeb.12490

[brv12534-bib-0262] * Shama, L. N. S. , Strobel, A. , Mark, F. C. & Wegner, K. M. (2014). Transgenerational plasticity in marine sticklebacks: maternal effects mediate impacts of a warming ocean. Functional Ecology 28, 1482–1493.

[brv12534-bib-0263] Shapiro, M. D. , Marks, M. E. , Peichel, C. L. , Blackman, B. K. , Nereng, K. S. , Jonsson, B. , Schluter, D. & Kingsley, D. M. (2004). Genetic and developmental basis of evolutionary pelvic reduction in threespine sticklebacks. Nature 428, 717–723. 15085123 10.1038/nature02415

[brv12534-bib-0264] Siepielski, A. M. , DiBattista, J. D. & Carlson, S. M. (2009). It's about time: the temporal dynamics of phenotypic selection in the wild. Ecology Letters 12, 1261–1276. 19740111 10.1111/j.1461-0248.2009.01381.x

[brv12534-bib-0265] Siwertsson, A. , Knudsen, R. , Kahilainen, K. K. , Præbel, K. , Primicerio, R. & Amundsen, P. A. (2010). Sympatric diversification as influenced by ecological opportunity and historical contingency in a young species lineage of whitefish. Evolutionary Ecology Research 12, 929–947.

[brv12534-bib-0266] Skúlason, S. & Smith, T. B. (1995). Resource Polymorphisms in Vertebrates. Trends in Ecology & Evolution 10, 366–370. 21237070 10.1016/s0169-5347(00)89135-1

[brv12534-bib-0267] * Skúlason, S. , Noakes, D. L. G. & Snorrason, S. S. (1989a). Ontogeny of trophic morphology in 4 sympatric morphs of arctic charr *Salvelinus alpinus* in Thingvallavatn, Iceland. Biological Journal of the Linnean Society 38, 281–301.

[brv12534-bib-0268] Skúlason, S. , Snorrason, S. S. & Jónsson, B. (1999). Sympatric morphs, populations and speciation in freshwater fish with an emphasis on Arctic charr In Evolution of Biological Diversity (eds Magurran A. E. and May R. M.), pp. 70–92. Oxford University Press, Oxford.

[brv12534-bib-0269] * Skúlason, S. , Snorrason, S. S. , Noakes, D. L. G. , Ferguson, M. M. & Malmquist, H. J. (1989b). Segregation in spawning and early life‐history among polymorphic Arctic charr, *Salvelinus alpinus*, in Thingvallavatn, Iceland. Journal of Fish Biology 35, 225–232.

[brv12534-bib-0270] Smith, T. B. (1993). Disruptive selection and the genetic‐basis of bill size polymorphism in the African finch *Pyrenestes* . Nature 363, 618–620.

[brv12534-bib-0271] Smith, G. & Ritchie, M. G. (2013). How might epigenetics contribute to ecological speciation? Current Zoology 59, 686–696.

[brv12534-bib-0272] Smith, T. B. & Skúlason, S. (1996). Evolutionary significance of resource polymorphisms in fishes, amphibians, and birds. Annual Review of Ecology and Systematics 27, 111–133.

[brv12534-bib-0273] Smith, G. , Smith, C. , Kenny, J. G. , Chaudhuri, R. R. & Ritchie, M. G. (2015). Genome‐wide DNA methylation patterns in wild samples of two morphotypes of threespine stickleback (*Gasterosteus aculeatus*). Molecular Biology and Evolution 32, 888–895. 25534027 10.1093/molbev/msu344

[brv12534-bib-0274] Smith, G. R. , Rettig, J. E. , Mittelbach, G. G. , Valiulis, J. L. & Schaack, S. R. (1999). The effects of fish on assemblages of amphibians in ponds: a field experiment. Freshwater Biology 41, 829–837.

[brv12534-bib-0275] Smith, T. A. , Martin, M. D. , Nguyen, M. & Mendelson, T. C. (2016). Epigenetic divergence as a potential first step in darter speciation. Molecular Ecology 25, 1883–1894. 26837057 10.1111/mec.13561

[brv12534-bib-0276] Snorrason, S. S. & Skúlason, S. (2004). Adaptive speciation in northern freshwater fish – patterns and processes In Adaptive Speciation (eds Dieckmann U., Doebeli M., Metz J. A. J. and Tautz D.), pp. 210–228. Cambridge University Press, Cambridge.

[brv12534-bib-0277] Snorrason, S. S. , Jónasson, P. M. , Jonsson, B. , Lindem, T. , Malmquist, H. J. , Sandlund, O. T. & Skúlason, S. (1992). Population‐dynamics of the planktivorous arctic charr, *Salvelinus alpinus* ("murta") in Thingvallavatn. Oikos 64, 352–364.

[brv12534-bib-0278] * Snorrason, S. S. , Skúlason, S. , Jonsson, B. , Malmquist, H. J. , Jónasson, P. M. , Sandlund, O. T. & Lindem, T. (1994). Trophic specialization in Arctic charr, *Salvelinus alpinus* (Pisces, Salmonidae) ‐ morphological divergence and ontogenetic niche shifts. Biological Journal of the Linnean Society 52, 1–18.

[brv12534-bib-0279] Sultan, S. E. (2015). Organism & Environment. Ecological Development, Niche Construction and Adaptation. Oxford Univerity Press, New York.

[brv12534-bib-0280] Sultan, S. E. & Spencer, H. G. (2002). Metapopulation structure favors plasticity over local adaptation. American Naturalist 160, 271–283. 10.1086/341015 18707492

[brv12534-bib-0281] Svanbäck, R. & Eklöv, P. (2003). Morphology dependent foraging efficiency in perch: a trade‐off for ecological specialization? Oikos 102, 273–284.

[brv12534-bib-0282] Svanbäck, R. & Eklöv, P. (2004). Morphology in perch affects habitat specific feeding efficiency. Functional Ecology 18, 503–510.

[brv12534-bib-0283] Svanbäck, R. & Eklöv, P. (2006). Genetic variation and phenotypic plasticity: causes of morphological and dietary variation in Eurasian perch. Evolutionary Ecology Research 8, 37–49.

[brv12534-bib-0284] Svanbäck, R. & Eklöv, P. (2011). Catch me if you can ‐ predation affects divergence in a polyphenic species. Evolution 65, 3515–3526. 22133222 10.1111/j.1558-5646.2011.01398.x

[brv12534-bib-0285] Svanbäck, R. & Persson, L. (2009). Population density fluctuations change the selection gradient in Eurasian perch. American Naturalist 173, 507–516. 10.1086/597223 19226234

[brv12534-bib-0286] Svanbäck, R. & Schluter, D. (2012). Niche specialization influences adaptive phenotypic plasticity in the threespine stickleback. American Naturalist 180, 50–59. 10.1086/666000 22673650

[brv12534-bib-0287] * Svanbäck, R. , Eklöv, P. , Fransson, R. & Holmgren, K. (2008). Intraspecific competition drives multiple species resource polymorphism in fish communities. Oikos 117, 114–124.

[brv12534-bib-0288] Svanbäck, R. , Pineda‐Krch, M. & Doebeli, M. (2009). Fluctuating population dynamics promotes the evolution of phenotypic plasticity. American Naturalist 174, 176–189. 10.1086/600112 19519278

[brv12534-bib-0289] Svensson, E. I. (2018). On reciprocal causation in the evolutionary process. Evolutionary Biology 1, 1–14. 10.1007/s11692-017-9431-x PMC5816131 29497217

[brv12534-bib-0290] * Swanson, H. K. , Kidd, K. A. , Babaluk, J. A. , Wastle, R. J. , Yang, P. P. , Halden, N. M. & Reist, J. D. (2010). Anadromy in Arctic populations of lake trout (*Salvelinus namaycush*): otolith microchemistry, stable isotopes, and comparisons with Arctic char (*Salvelinus alpinus*). Canadian Journal of Fisheries and Aquatic Sciences 67, 842–853.

[brv12534-bib-0291] * Taylor, E. B. (1999). Species pairs of north temperate freshwater fishes: evolution, taxonomy, and conservation. Reviews in Fish Biology and Fisheries 9, 299–324.

[brv12534-bib-0292] * Taylor, E. B. & Mcphail, J. D. (1999). Evolutionary history of an adaptive radiation in species pairs of threespine sticklebacks (*Gasterosteus*): insights from mitochondrial DNA. Biological Journal of the Linnean Society 66, 271–291.

[brv12534-bib-0293] Taylor, E. B. , Boughman, J. W. , Groenenboom, M. , Sniatynski, M. , Schluter, D. & Gow, J. L. (2006). Speciation in reverse: morphological and genetic evidence of the collapse of a three‐spined stickleback (*Gasterosteus aculeatus*) species pair. Molecular Ecology 15, 343–355. 16448405 10.1111/j.1365-294X.2005.02794.x

[brv12534-bib-0294] Thomas, S. M. , Harrod, C. , Hayden, B. , Malinen, T. & Kahilainen, K. K. (2017). Ecological speciation in a generalist consumer expands the trophic niche of a dominant predator. Scientific Reports 7, 8765. 28821736 10.1038/s41598-017-08263-9 PMC5562900

[brv12534-bib-0295] Thuiller, W. , Munkemuller, T. , Lavergne, S. , Mouillot, D. , Mouquet, N. , Schiffers, K. & Gravel, D. (2013). A road map for integrating eco‐evolutionary processes into biodiversity models. Ecology Letters 16, 94–105. 23679011 10.1111/ele.12104 PMC3790307

[brv12534-bib-0296] Tobler, M. , Kelley, J. L. , Plath, M. & Riesch, R. (2018). Extreme environments and the origins of biodiversity: adaptation and speciation in sulphide spring fishes. Molecular Ecology 27, 843–859. 29368386 10.1111/mec.14497

[brv12534-bib-0297] Townsend, C. R. , Sutherland, W. J. & Perrow, M. R. (1990). A modeling investigation of population‐cycles in the fish *Rutilus rutilus* . Journal of Animal Ecology 59, 469–485.

[brv12534-bib-0298] Tufto, J. (2015). Genetic evolution, plasticity, and bet‐hedging as adaptive responses to temporally autocorrelated fluctuating selection: a quantitative genetic model. Evolution 69, 2034–2049. 26140293 10.1111/evo.12716

[brv12534-bib-0299] * Turgeon, J. & Bernatchez, L. (2001). Clinal variation at microsatellite loci reveals historical secondary intergradation between glacial races of *Coregonus artedi* (Teleostei: Coregoninae). Evolution 55, 2274–2286. 11794787 10.1111/j.0014-3820.2001.tb00742.x

[brv12534-bib-0300] * Turgeon, J. & Bernatchez, L. (2003). Reticulate evolution and phenotypic diversity in North American ciscoes, *Coregonus* ssp (Teleostei: Salmonidae): implications for the conservation of an evolutionary legacy. Conservation Genetics 4, 67–81.

[brv12534-bib-0301] * Turgeon, J. , Estoup, A. & Bernatchez, L. (1999). Species flock in the North American Great lakes: molecular ecology of Lake Nipigon Ciscoes (Teleostei : Coregonidae : Coregonus). Evolution 53, 1857–1871. 28565465 10.1111/j.1558-5646.1999.tb04568.x

[brv12534-bib-0302] Uller, T. , Moczek, A. P. , Watson, R. A. , Brakefield, P. M. & Laland, K. N. (2018). Developmental bias and evolution: a regulatory network perspective. Genetics 209, 949–966. 30049818 10.1534/genetics.118.300995 PMC6063245

[brv12534-bib-0303] Van Valen, L. (1973). Festschrift. Science 180, 488.

[brv12534-bib-0304] * Vecsei, P. , Blackie, C. T. , Muir, A. M. , Machtans, H. M. & Reist, J. D. (2012). A preliminary assessment of cisco (*Coregonus* spp.) diversity in Yellowknife Bay, Great Slave Lake, Northwest Territories In Biology and Management of Coregonid Fishes ‐ 2008 *Advances in Limnology* (Volume 63, eds Tallman R. F., Howland K. L., Rennie R. D. and Mills K.), pp. 299–322. E Schweizerbart'sche Verlagsbuchhandlung, Stuttgart.

[brv12534-bib-0305] Via, S. (2009). Natural selection in action during speciation. Proceedings of the National Academy of Sciences of the United States of America 106, 9939–9946. 19528641 10.1073/pnas.0901397106 PMC2702801

[brv12534-bib-0306] Vindenes, Y. & Langangen, O. (2015). Individual heterogeneity in life histories and eco‐evolutionary dynamics. Ecology Letters 18, 417–432. 25807980 10.1111/ele.12421 PMC4524410

[brv12534-bib-0307] Vonlanthen, P. , Bittner, D. , Hudson, A. G. , Young, K. A. , Muller, R. , Lundsgaard‐Hansen, B. , Roy, D. , Di Piazza, S. , Largiader, C. R. & Seehausen, O. (2012). Eutrophication causes speciation reversal in whitefish adaptive radiations. Nature 482, 357–362. 22337055 10.1038/nature10824

[brv12534-bib-0308] * Vonlanthen, P. , Roy, D. , Hudson, A. G. , Largiader, C. R. , Bittner, D. & Seehausen, O. (2009). Divergence along a steep ecological gradient in lake whitefish (*Coregonus* sp.). Journal of Evolutionary Biology 22, 498–514. 19170819 10.1111/j.1420-9101.2008.01670.x

[brv12534-bib-0309] Waddington, C. H. (1942). Canalization of development and the inheritance of acquired characters. Nature 150, 563–565. 10.1038/1831654a0 13666847

[brv12534-bib-0310] Waddington, C. H. (1957a). The genetic basis of 'assimilated bithorax' stock. Journal of Genetics 55, 241–245. 10.1007/BF02729015 17072078

[brv12534-bib-0311] Waddington, C. H. (1957b). The Strategy of the Genes: A Discussion of Some Aspects of Theoretical Biology. Allen & Unwin, Sydney.

[brv12534-bib-0312] Wainwright, P. C. , Osenberg, C. W. & Mittelbach, G. G. (1991). Trophic polymorphism in the pumpkinseed sunfish (*Lepomis‐Gibbosus* Linnaeus) ‐ effects of environment on ontogeny. Functional Ecology 5, 40–55.

[brv12534-bib-0313] Walsh, M. R. , DeLong, J. P. , Hanley, T. C. & Post, D. M. (2012). A cascade of evolutionary change alters consumer‐resource dynamics and ecosystem function. Proceedings of the Royal Society B‐Biological Sciences 279, 3184–3192. 10.1098/rspb.2012.0496 PMC3385726 22628469

[brv12534-bib-0314] Ward, J. M. & Ricciardi, A. (2007). Impacts of *Dreissena* invasions on benthic macroinvertebrate communities: a meta‐analysis. Diversity and Distributions 13, 155–165.

[brv12534-bib-0315] * Weese, D. J. , Ferguson, M. M. & Robinson, B. W. (2012). Contemporary and historical evolutionary processes interact to shape patterns of within‐lake phenotypic divergences in polyphenic pumpkinseed sunfish, *Lepomis gibbosus* . Ecology and Evolution 2, 574–592. 22822436 10.1002/ece3.72 PMC3399146

[brv12534-bib-0316] Weis, J. J. & Post, D. M. (2013). Intraspecific variation in a predator drives cascading variation in primary producer community composition. Oikos 122, 1343–1349.

[brv12534-bib-0317] West‐Eberhard, M. J. (2003). Developmental Plasticity and Evolution. Oxford University Press, Oxford.

[brv12534-bib-0318] West‐Eberhard, M. J. (2005). Phenotypic accommodation: adaptive innovation due to developmental plasticity. Journal of Experimental Zoology Part B‐Molecular and Developmental Evolution 304B, 610–618. 10.1002/jez.b.21071 16161068

[brv12534-bib-0319] * Wilson, A. J. , Gíslason, D. , Skúlason, S. , Snorrason, S. S. , Adams, C. E. , Alexander, G. , Danzmann, R. G. & Ferguson, M. M. (2004). Population genetic structure of Arctic Charr, *Salvelinus alpinus* from northwest Europe on large and small spatial scales. Molecular Ecology 13, 1129–1142. 15078451 10.1111/j.1365-294X.2004.02149.x

[brv12534-bib-0320] * Wilson, D. S. , Muzzall, P. M. & Ehlinger, T. J. (1996). Parasites, morphology, and habitat use in a bluegill sunfish (*Lepomis macrochirus*) population. Copeia 2, 348–354.

[brv12534-bib-0321] Wimberger, P. H. (1994). Trophic polymorphism, plasticity and speciation in vertebrates In Advances in Fish Foraging Theory (eds Stouder D. J. and Fresh K.). Belle Baruch Press, Columbia.

[brv12534-bib-0322] * Witt, J. D. S. , Zemlak, R. J. & Taylor, E. B. (2011). Phylogeography and the origins of range disjunctions in a north temperate fish, the pygmy whitefish (*Prosopium coulterii*), inferred from mitochondrial and nuclear DNA sequence analysis. Journal of Biogeography 38, 1557–1569.

[brv12534-bib-0323] Wolinsky, E. & Libby, E. (2016). Evolution of regulated phenotypic expression during a transition to multicellularity. Evolutionary Ecology 30, 235–250.

[brv12534-bib-0324] * Woods, P. J. , Muller, R. & Seehausen, O. (2009). Intergenomic epistasis causes asynchronous hatch times in whitefish hybrids, but only when parental ecotypes differ. Journal of Evolutionary Biology 22, 2305–2319. 19824932 10.1111/j.1420-9101.2009.01846.x

[brv12534-bib-0325] Woods, P. J. , Skúlason, S. , Snorrason, S. S. , Kristjánsson, B. K. , Malmquist, H. J. & Quinn, T. P. (2012a). Intraspecific diversity in Arctic charr, *Salvelinus alpinus*, in Iceland: I. Detection using mixture models. Evolutionary Ecology Research 14, 973–992.

[brv12534-bib-0326] Woods, P. J. , Skúlason, S. , Snorrason, S. S. , Kristjánsson, B. K. , Malmquist, H. J. & Quinn, T. P. (2012b). Intraspecific diversity in Arctic charr, *Salvelinus alpinus*, in Iceland: II. Which environmental factors influence resource polymorphism in lakes? Evolutionary Ecology Research 14, 993–1013.

[brv12534-bib-0327] Wund, M. A. , Baker, J. A. , Clancy, B. , Golub, J. L. & Fosterk, S. A. (2008). A test of the "Flexible stem" model of evolution: ancestral plasticity, genetic accommodation, and morphological divergence in the threespine stickleback radiation. American Naturalist 172, 449–462. 10.1086/590966 18729721

[brv12534-bib-0328] Wund, M. A. , Valena, S. , Wood, S. & Baker, J. A. (2012). Ancestral plasticity and allometry in threespine stickleback reveal phenotypes associated with derived, freshwater ecotypes. Biological Journal of the Linnean Society 105, 573–583. 22611287 10.5061/dryad.hb824gd4 PMC3351840

[brv12534-bib-0329] Young, R. L. & Badyaev, A. V. (2010). Developmental plasticity links local adaptation and evolutionary diversification in foraging morphology. Journal of Experimental Zoology Part B: Molecular and Developmental Evolution 314B, 434–444. 10.1002/jez.b.21349 20700888

[brv12534-bib-0330] * Zimmerman, M. S. , Schmidt, S. N. , Krueger, C. C. , Vander Zanden, M. J. & Eshenroder, R. L. (2009). Ontogenetic niche shifts and resource partitioning of lake trout morphotypes. Canadian Journal of Fisheries and Aquatic Sciences 66, 1007–1018.

