# Supplementary material for: A way forward with eco evo devo: an extended theory of resource polymorphism with postglacial fishes as model systems
Source: Biol Rev Camb Philos Soc. 2019 Jun 19;94(5):1786–808. doi: 10.1111/brv.12534 (PMC6852119; doi:10.1111/brv.12534)
Supplement: Supplementary file 1 — Table S1. Examples of empirical evidence for different patterns of resource polymorphism in postglacial freshwater fishes including phenotypic distribution, trait type, ecological axis of divergence, and evidence of reproductive isolation. Appendix S1. Overview of resource polymorphism in fish in postglacial freshwater systems. [file BRV-94-1786-s001.docx]

**Table S1.** Examples of empirical evidence for different patterns of resource polymorphism in postglacial freshwater fishes, including phenotypic distribution, trait type, ecological axis of divergence, and evidence of reproductive isolation. Species were chosen to represent different major orders of freshwater fish as well as representing well-studied species.

Study Phenotypic Type (and number) Axis of Evo-devo Reproductive

species distribution of phenotypes divergence basis of phenotype Eco-Evo Isolation (References)

**CYPRINIFORMES**

**(cyprinids)**

Roach Uni, Disc, Bi MP (1–2) LI-PE unknown C Unknown 1, 2

(*Rutilus rutilus*) *F_ST_* 0–0.134

**GASTEROSTEIFORMES**

**(sticklebacks)**

Three-spined stickleback Uni, Disc, Bi, Sep MP, LH (1–2) LI-LI, LI-PE, E, G C, Pr, V, Pa, HC Spawn 3–15

(*Gasterosteus aculeatus*) STR-LA, STR-SEA *F_ST_* 0–0.08

**PERCIFORMES (percids)**

Eurasian perch Uni, Disc, Bi MP (1–2) LI-PE E – 69% C, Pr, V Unknown 1, 2, 16–21

(*Perca fluviatilis*) G – <2% *F_ST_* 0–0.17

Bluegill sunfish Uni, Disc, Bi MP (1–2) LI-PE unknown C, Pr, Pa Unknown 22–25

(*Lepomis macrochirus*)

Pumpkinseed sunfish Uni, Disc, Bi MP (1–2) LI-PE E – 53% C Spawn 26–33

(*Lepomis gibbosus*) G – 14% *F_ST_* 0–0.005

**OSMERIFORMES**

**(osmerids)**

Rainbow smelt Uni, Disc, Bi MP, LH (1–3) PEv, STR-SEA unknown C, HC? Spawn (t, s) 34–38

(*Osmerus mordax*) *F_ST_* 0.015–0.234

**SALMONIFORMES (salmonids)**

**– subfamily Salmoninae**

Arctic charr Uni, Disc, Bi, Sep MP, LH (1–4) LI-PE-Pro, STR-SEA E, G, M C, Pr, Pa, LS Spawn (t, s) 39–46

(*Salvelinus alpinus*) *F_ST_* 0.056–0.381

Brook charr Uni, Disc MP, LH (1–2) LI-PE, STR-SEA E, G C unknown 47–49

(*Salvelinus fontinalis*)

Lake charr Uni, Disc, Bi MP, LH (1–4) LI-PE-Pro, PEv, unknown? C, P *F_ST_* 0–0.071 50–55

(*Salvelinus namaycush*) STR-SEA

**SALMONIFORMES**

**(salmonids) – subfamily Coregoninae**

Lake whitefish Uni, Disc, Bi, Sep MP, LH, PH (1–2) LI-PE E, G, M C, Pa, HC Spawn (t, s, PZ) 56–61

(*Coregonus clupeaformis*) *F_ST_* 0–0.256

Whitefish Uni, Disc, Bi, Sep MP, LH (1–5) LI-PE-Pro, PEv, E, G C, Pr, Pa, V, LS, HC Spawn (t, s) 62–73

(*Coregonus lavaretus*) STR-LA, STR-SEA *F_ST_* 0–0.153

North American ciscoes Uni, Disc, Bi, Sep MP (1–7) PEv, STR-LA E, G C, HC unknown 74–78

(*Coregonus* spp.) *F_ST_* 0–0.08

Vendace Uni, Disc, Bi MP, LH, PH (1–2) PEv G HC Spawn (t, s) 79–83

(*Coregonus albula*) F_ST_ 0-0.039

Pygmy whitefish Uni, Disc, Bi MP (1–3) LI-PE, LI-PE-Pro unknown C Unknown 84–86

(*Prosopium* *coulterii*) *F_ST_* 0–0.025

Phenotypic distribution: Uni, unimodal; Disc, discontinuous; Bi, bimodal; Sep, separate.

Type of phenotype: MP, morphological; LH, life history; PH, physiological. For life-history phenotypes we mean traits such as age and size at maturation, or migratory *versus* resident. It is likely that behavioural differences are also important, especially in the early stages of divergence.

Axis of divergence: LI-PE, littoral–pelagic axis; LI-PE-Pro, littoral–pelagic–profundal; PEv, pelagic vertical; STR-LA, stream–lake; STR-SEA, stream–sea.

Basis of phenotype: E, environmental; G, genetic; M, maternal. This is based on results from common garden experiments and percentages are statistical estimates from these experiments.

Eco-Evo: C, intra-specific competition; Pr, predation; Pa, parasites; V, visual conditions or productivity; LS, lake size or depth; HC, historical contingency. This column describes the ecological drivers of divergent natural selection.

Reproductive isolation – evidence of reproductive isolation (or lack of reproductive isolation): F_ST_, measured genetic divergence between forms; Spawn, temporal (t) or spatial (s) separation in spawning; PZ, post-zygotic isolation.

References: 1, Svanbäck *et al*. (2008); 2, Faulks *et al.* (2015); 3, Svanbäck & Schluter (2012); 4, Ingram *et al*. (2012); 5, Schluter (1993); 6, Schluter (1995); 7, Day *et al*. (1994); 8, McPhail (1984); 9, Taylor & McPhail (1999); 10, Schluter & McPhail (1992); 11, Hendry *et al*. (2009); 12, Kristjánsson *et al*. (2002*a*); 13, Ólafsdóttir *et al*. (2006); 14, Ólafsdóttir *et al*. (2007*a*); 15, Ólafsdóttir *et al*. (2007*b*); 16, Svanbäck & Eklöv (2003); 17, Svanbäck & Eklöv (2006); 18, Eklöv & Svanbäck (2006); 19, Quevedo *et al*. (2009); 20, Bartels *et al*. (2012); 21, Hirsch *et al*. (2013*b*); 22, Ehlinger (1990); 23, Ehlinger & Wilson (1988); 24, Wilson *et al*. (1996); 25, Chipps *et al*. (2004); 26, Weese *et al*. (2012); 27, Jastrebski & Robinson (2004); 28, Robinson & Wilson (1996); 29, Robinson *et al*. (1993); 30, Robinson *et al*. (1996); 31, Robinson *et al*. (2000); 32, McCairns & Fox (2004); 33, Colborne *et al*. (2016); 34, Curry *et al*. (2004); 35, Saint-Laurent *et al*. (2003); 36, Bradbury *et al*. (2010); 37, Barrette *et al*. (2009); 38, Bernantchez (1997); 40, Skúlason *et al*. (1989); 41, Gíslason *et al*. (1999); 42, Skúlason *et al*. (1999); 43, Adams & Huntingford (2002); 44, Wilson *et al*. (2004); 45, Klemetsen (2010); 46, Parsons *et al*. (2011); 47, Crespel *et al*. (2017); 48, Bourke *et al*. (1997); 49, Proulx & Magnan (2004); 50, Swanson *et al*. (2010); 51, Moore & Bronte (2001); 52, Zimmerman *et al*. (2009); 53, Hansen *et al*. (2012); 54, Chavarie *et al*. (2013); 55, Perreault-Payette *et al*. (2017); 56, Bernatchez (2004); 57, Bernatchez *et al*. (1999); 58, Bernatchez *et al*. (2010); 59, Lu & Bernatchez (1999); 60, Pavey *et al*. (2013); 61, Rogers & Bernatchez (2006); 62, Harrod *et al*. (2010); 63, Kahilainen & Lehtonen (2002); 64, Kahilainen *et al*. (2011); 65, Knudsen *et al*. (2003); 66, Østbye *et al*. (2006); 67, Østbye *et al*. (2005); 68, Præbel *et al*. (2013); 69, Siwertsson *et al*. (2010); 70, Vonlanthen *et al*. (2009); 71, Woods *et al*. (2009); 72, Hudson *et al*. (2011); 73, Ozerov *et al*. (2016); 74, Turgeon *et al*. (1999); 75, Turgeon & Bernatchez (2001); 76, Turgeon & Bernatchez (2003); 77, Vecsei *et al*. (2012); 78, Eshenroder *et al*. (2016); 79, Schulz *et al*. (2006); 80, Helland *et al*. (2008); 81, Helland *et al*. (2009); 82, Ohlberger *et al*. (2008); 83, Delling *et al*. (2014); 84, McCart (1970); 85, Gowell *et al*. (2012); 86, Witt *et al*. (2011).

**Appendix S1. Overview of resource polymorphism in fish in postglacial freshwater systems.**

The phenotypic and ecological segregation of resource-polymorphic fishes in postglacial lakes is predominantly along either littoral–pelagic, littoral–profundal or a combination of all three resource axes. This separation along resource gradients reflects a trade-off in foraging efficiency, whereby deeper bodied individuals are better at foraging in the structured littoral or profundal habitat compared to more streamlined individuals, whereas the opposite pattern is true in the pelagic (limnetic) habitat (Ehlinger & Wilson, 1988; Sandlund *et al.*, 1992; Schluter, 1995; Svanbäck & Eklöv, 2003, 2004; Kahilainen & Østbye, 2006). Other adaptations to these different feeding habitats relate to body/head size and shape, mouth position, gill raker density and length – all traits that influence feeding efficiency in specific habitats. In particular, a small body size, a long pointed head with a terminal mouth and numerous long gill rakers are typically found amongst morphs targeting small-sized prey in the pelagic zone, whereas the opposite often holds true for benthic morphs (Byström & Andersson, 2005). Although the littoral–pelagic axis of divergence is common, other forms of resource-based divergence in postglacial fishes also occur, such as divergence between different benthic habitats [e.g. mud *versus* lava in volcanic lakes (Snorrason *et al.*, 1994; Kristjánsson *et al*., 2002*a*); Table S1] and between lake–stream habitats (Hendry *et al*., 2002; Table S1).

An important observation is that the degree of resource-based phenotypic segregation varies both among and within species (Table S1), suggesting a continuum in the process of divergence. Parallel phenotypic and ecological segregation has been identified repeatedly within and across species (Fig. 2; Table S1). Hence these systems provide powerful models to elucidate the mechanisms of divergence through comparative studies (Skúlason *et al*., 1999; Snorrason & Skúlason, 2004; Hendry *et al.*, 2009). Empirical case studies on a small subset of systems indicate that the magnitude of phenotypic divergence reflects variation in the levels of recent gene flow (e.g. Wilson *et al.*, 2004; Østbye *et al.*, 2006), the magnitude of environmental differences (Kaeuffer *et al.*, 2012) and the relative contribution of phenotypic plasticity (Parsons *et al.*, 2011; Oke *et al.*, 2016). Hence, an increased understanding of the interacting roles of gene flow and natural selection in adaptive diversification and speciation has been achieved (e.g. Räsänen & Hendry, 2008; Hendry *et al.*, 2009; Seehausen & Wagner, 2014) and the role of historical contingency (e.g. colonization history) is also recognized (Gíslason *et al.*, 1999; Taylor, 1999; Bernatchez *et al.*, 2010; Kapralova *et al.*, 2011).
